# Towards a neutron and X-ray reflectometry environment for the study of solid–liquid interfaces under shear

**DOI:** 10.1038/s41598-021-89189-1

**Published:** 2021-05-06

**Authors:** Alexander J. Armstrong, Thomas M. McCoy, Rebecca J. L. Welbourn, Robert Barker, Jonathan L. Rawle, Beatrice Cattoz, Peter J. Dowding, Alexander F. Routh

**Affiliations:** 1 BP Institute and Department of Chemical Engineering and Biotechnology, University of Cambridge, Cambridge, UK; 2 ISIS Neutron and Muon Source, Didcot, UK; 3 School of Physical Sciences, University of Kent, Ingram Building, Canterbury, UK; 4 Diamond Light Source Ltd, Diamond House, Harwell Campus, Didcot, OX11 0DE UK; 5 Infineum UK Ltd, Milton Hill, UK

**Keywords:** Characterization and analytical techniques, Surface assembly

## Abstract

A novel neutron and X-ray reflectometry sample environment is presented for the study of surface-active molecules at solid–liquid interfaces under shear. Neutron reflectometry was successfully used to characterise the iron oxide–dodecane interface at a shear rate of $7.0\times {}10^{2}$ $\hbox {s}^{-1}$ using a combination of conventional reflectometry theory coupled with the summation of reflected intensities to describe reflectivity from thicker films. Additionally, the structure adopted by glycerol monooleate (GMO), an Organic Friction Modifier, when adsorbed at the iron oxide–dodecane interface at a shear rate of $7.0\times {}10^{2}$ $\hbox {s}^{-1}$ was studied. It was found that GMO forms a surface layer that appears unaltered by the effect of shear, where the thickness of the GMO layer was found to be $24.3^{+9.9}_{-10.2}$ Å under direct shear at $7.0\times {}10^{2}$ $\hbox {s}^{-1}$ and $25.8^{+4.4}_{-5.2}$ Å when not directly under shear. Finally, a model to analyse X-ray reflectometry data collected with the sample environment is also described and applied to data collected at $3.0\times {}10^{3}$ $\hbox {s}^{-1}$.

## Introduction

The behaviour of surface-active molecules (surfactants) at solid–liquid interfaces is of key importance to applications such as detergency, froth flotation and lubrication. In such processes adsorbed additives are subject to shear from the surrounding fluid, where the shear rate dictates the extent to which adsorbate molecules are perturbed from their adsorbed structure under static conditions. Shear rates across these example applications are estimated to vary between 10$^{2}$–10$^{8}$ $\hbox {s}^{-1}$^1,2^.

Organic Friction Modifiers (OFMs) are surfactants included in engine oil formulations to reduce frictional losses in high-pressure tribological contacts that arise between mechanical engine components. The conventional understanding is that OFMs adsorb at metallic engine surfaces through the interaction of the polar head group with the hydrophilic engine surface while the hydrophobic alkyl chain extends into the bulk hydrocarbon solvent. Compact surface layers are formed as a result of van der Waals attractions between adjacent molecules. When layers on opposing moving surfaces are brought into contact, slip-planes are formed that reduce the frictional force^3^. This mechanistic understanding has been developed over the twentieth century where the majority of researchers used tribometer friction tests and adsorption studies^4–8^.

More recent studies have used scanning probe microscopy and surface-force apparatus to study the structure and nanotribology of surface films^9–11^. Results from atomic force microscopy suggest OFMs self-assemble at steel-hydrocarbon interfaces, forming monolayer surface films^12–14^. Specialised tribometers equipped with interferometric microscopes have been used to characterise the in-situ formation of OFM monolayer films in tribological contacts^15^. However, these experiments have also revealed film thicknesses beyond monolayer length scales for self-assembled fatty acids at steel surfaces^16,17^. Furthermore, it has been shown that fatty acids dispersed in organic solvent with trace amounts of water can form films thicker than monolayers at steel surfaces^18^. Findings such as these indicate that friction-reducing film structures are not limited to monolayers and indeed, the suggestion of thicker boundary films has been debated previously^19^. Whilst it is established that deposited monolayer structures can reduce friction between flat surfaces^20^, there is less evidence to suggest that self-assembled OFM interfacial structures are monolayers. The fundamental mechanism behind friction modification can be elucidated by understanding how OFMs self-assemble at interfaces and how their surface structure varies with applied tribological conditions.

The experimental determination of in-situ OFM friction-reducing surface structures is difficult as it requires the combination of a surface-specific technique with the harsh conditions applied within an engine. A major challenge in the development of such equipment is ensuring the detecting technique can probe the buried interface without perturbation and without significant interference from operating under tribological conditions. In this regard two candidate techniques are specular neutron and X-ray reflectometry (NR and XRR) which have been used to study the shear-dependent behaviour of molecules at solid–liquid interfaces^21,22^. While both are scattering methodologies that can measure the thicknesses and atomic densities of thin films perpendicular to an interface, there are some distinct differences between the two techniques. NR typically requires a greater sample area compared to XRR because the flux of neutron sources is lower than those available at X-ray synchrotrons. Despite this, NR is generally preferred for the study of buried solid–liquid interfaces as the penetration depth of neutrons is significantly greater. NR is further suited for the study of light-mass elements, especially hydrogen, found in most organic molecules, while XRR is more sensitive to heavier, electron-rich elements. Consequently, NR has been favoured for the study of polymers and surfactants at solid–liquid interfaces^23^.

Developments in NR sample environments have enabled the study of organic media at solid–liquid interfaces under various shear conditions. Perhaps the most user-friendly are closed-loop flow cells, which have been used to study surfactant shear-induced behaviour at the Si-water interface under laminar flow conditions^24,25^. Whilst shear rates have been reported to reach up to $5.5\times {}10^{4}$ $\hbox {s}^{-1}$ in Poiseuille shear cells^26^, it is difficult to control the range of accessible shear rates and it is not possible to study the elastic and loss shear moduli of adsorbed samples. Cone and plate rheometers have been fitted onto NR environments to accommodate these needs, enabling the application of oscillatory and steady shear to solid–liquid interfaces^27,28^.

Confinement cells have also been developed to study how adsorbate structure changes with applied pressure. Here, the main principle is to move a surface towards the interface of interest and to apply a pressure. This has been achieved by either the actuation of a solid surface towards the interface of interest or by the expansion of an inflatable elastic material against the interface^29–31^. While the former technique is complicated in terms of ensuring both surfaces remain parallel over the areas required for NR, shear is perhaps more trivial to apply at the interface with this type of confinement. Steady shear rates of up to 20 $\hbox {s}^{-1}$ and oscillatory shear rates up to $10^{4}$ $\hbox {s}^{-1}$ are reportedly possible in confinement cells^32^. By increasing either the flow rate or the surface velocity within a confinement cell it becomes possible to probe the interface at greater shear rates than previously achieved. This would enable the study of adsorbates under conditions more relevant to those found within tribological contacts.

**Figure 1:**
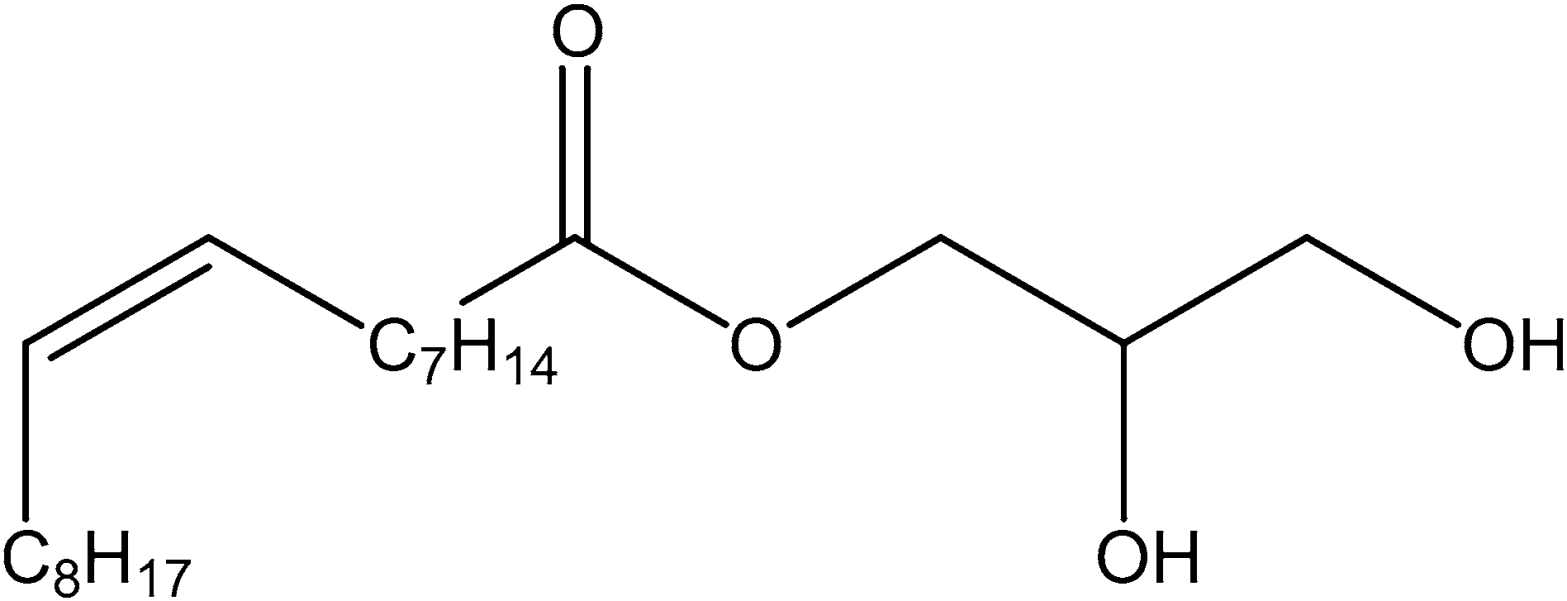
The molecular structure of glycerol monooleate (GMO).

In this paper a novel sample environment, referred to as the tribometer, is presented for the study of surface-active species under shear with NR and XRR. Initially, a model to describe the neutron reflectivity from an iron-coated silicon substrate with neat dodecane-$\hbox {d}_{26}$ entrained against the substrate surface is presented and discussed. NR is then used to characterise the self-assembled structure of glycerol monooleate (GMO), an industrially relevant OFM, adsorbed from solution at the iron oxide–dodecane interface at $7.0\times {}10^{2}$ $\hbox {s}^{-1}$. The molecular structure of GMO is shown in Fig. 1. Finally, XRR data collected with a 20 mM GMO-dodecane solution entrained against an iron-coated silicon substrate at a shear rate of $3.0\times {}10^{3}$ $\hbox {s}^{-1}$ is presented and analysed using a conventional slab model.

### Apparatus

**Figure 2:**
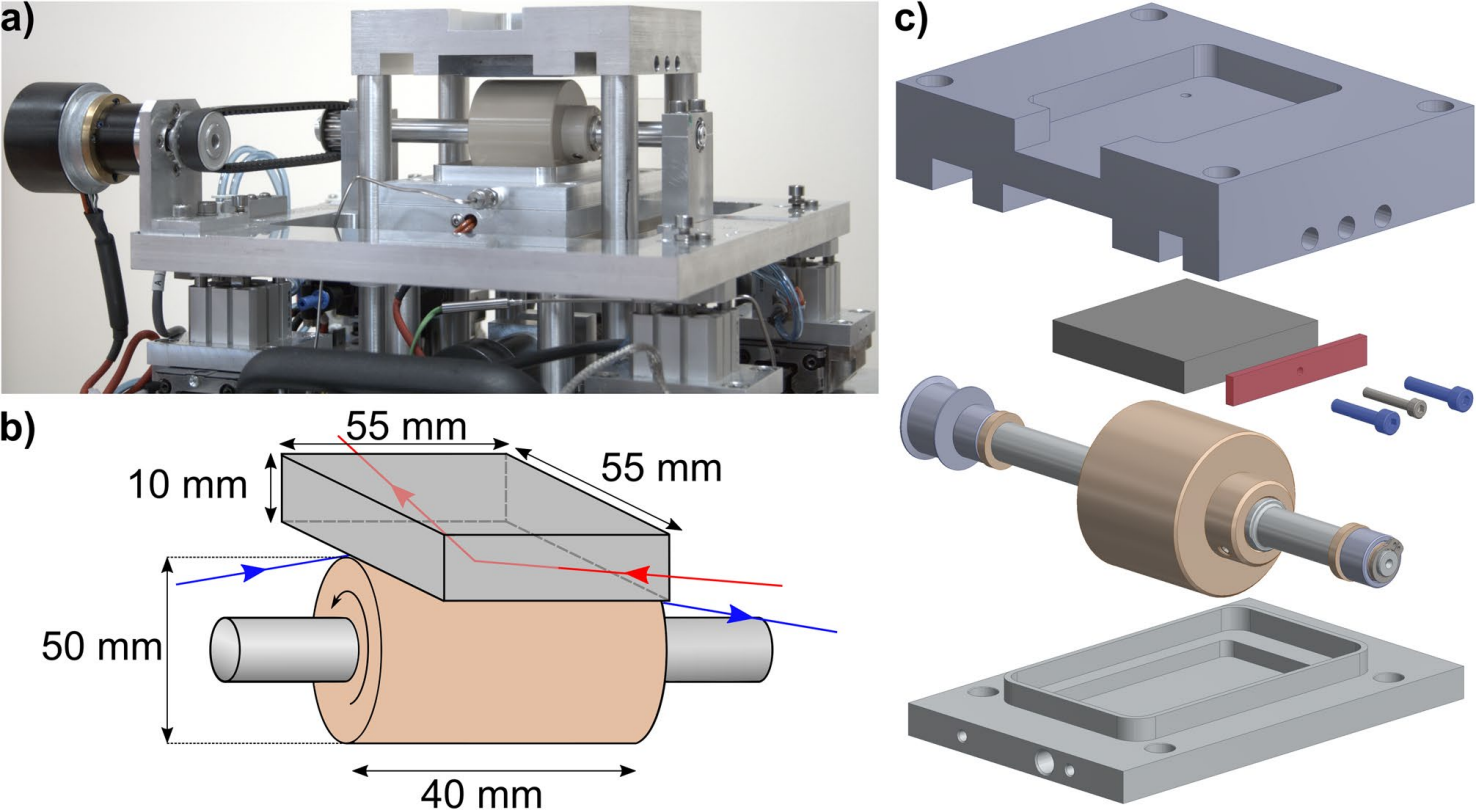
Visual overview of the tribometer. (**a**) Diagonal view of the tribometer in the lowered position. (**b**) A schematic of the roller and substrate at the centre of the tribometer. The red and blue arrows depict the approximate paths for the neutron and X-ray beams respectively. (**c**) Exploded view of the centre of the tribometer. The two screws (blue) in the head unit force the gib strip (red) against the substrate, securing it above the roller.

The tribometer, Fig. 2, was commissioned by Infineum UK Ltd and was manufactured by Cambridge Reactor Design, UK. It was designed to fit on both the INTER NR instrument at ISIS, UK and the FIGARO NR instrument at the Institut Laue-Langevin, France. It was also designed to fit on the I07 X-ray diffraction instrument at Diamond Light Source, UK.

**Figure 3:**
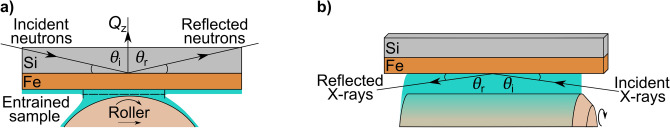
Principal of NR and XRR techniques with tribometer. (**a**) Schematic of NR setup with tribometer. In specular reflection the beam is reflected from the horizontal at an angle $\theta _{\mathrm {r}}$, where $\theta _{\mathrm {r}}$ = $\theta _{\mathrm {i}}$. The incident neutron beam illuminates an area that is greater than the meniscus region above the roller. The meniscus held above the roller is outlined by the dashed bracket in the schematic. (**b**) Schematic of XRR setup with tribometer. The incident X-ray beam strikes the horizontal dodecane-substrate interface at $\theta _{\mathrm {i}}$.

At the centre of the tribometer an aluminium shaft holds a polyether ether ketone (PEEK) roller partially submerged in an oil bath. Using a belt transmission system, the roller can be rotated with surface velocities between 1.4–7.2 $\times {}10^{-1}$ m $\hbox {s}^{-1}$. A 55 $\times$ 55 $\times$ 10 (l $\times$ w $\times$ h) mm substrate can be secured above the roller in a housing using two screws and a gib plate. The roller and motor sit on a rectangular aluminium plate that can be raised to either create a loaded contact or to form a specific gap between the roller and substrate. The former is facilitated by four pneumatic actuators that push the assembly plate upwards, producing a loaded contact between the roller and the substrate. The actuators are operated using compressed gas regulated between 0.3 bar and 4 bar, enabling varying loads through the contact of up to 120 N. Alternatively, the roller can be raised to a specific distance from the substrate by tightening four screws on the assembly plate. Using two laser displacement sensors (micro-epsilon ILD 1420s) the roller-substrate gap can be calibrated with micrometer precision. A second motor drives the aluminium plate along two parallel rails, allowing the reciprocation of the roller over 34 mm of the substrate’s surface at speeds up to $1.8\times {}10^{-3}$ m $\hbox {s}^{-1}$. At the end of each reciprocating stroke the rotation of the roller is reversed to match the direction of horizontal travel. The oil bath and substrate holder can be held at temperatures up to 120 $^{\circ }$C. All the operating electronics are controlled via a Eurotherm Mini8 controller. Schematics depicting the sample environment provided by the tribometer for NR and XRR are shown in Fig. 3a,b respectively.

## Results

### Neutron reflectometry

#### Operation

Iron-coated silicon substrates were loaded into the tribometer, and the roller-substrate gap was calibrated at 200 µm. The sample solutions were then entrained onto the substrate at one of two maximum shear rates: 7.0 $\times$ 10$^{2}$ $\hbox {s}^{-1}$ and 3.7 $\times$ 10$^{3}$ $\hbox {s}^{-1}$. The shear rates are calculated as the ratio of the roller surface velocity to the minimum roller-gap separation. The neutron beam was then aligned at the substrate-dodecane interface. Following this, the reflected intensity of neutrons was measured for at least 1.25 h. The minimum widths of the resulting menisci held between the roller and the substrate were measured post-experiment via calibrated photography; the widths were found to be 11.5 mm and 20.0 mm in the direction of the beam at 7.0 $\times$ 10$^{2}$ $\hbox {s}^{-1}$ and 3.7 $\times$ 10$^{3}$ $\hbox {s}^{-1}$, respectively. The greater meniscus width at the higher shear rate can be considered a result of increasing the angular velocity of the roller, since increasing the roller horizontal velocity had a negligible effect on the meniscus width. The region held outside of the meniscus remained visibly wetted by the solution.

#### Iron oxide–dodecane interface

**Figure 4:**
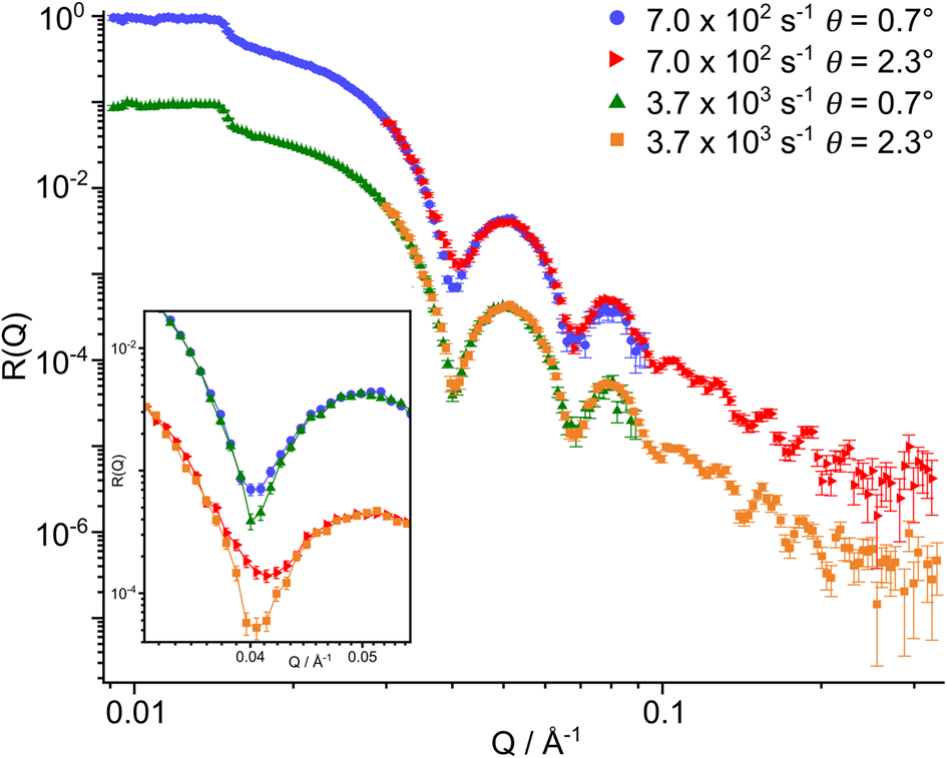
NR data for dodecane-$\hbox {d}_{26}$ entrained against an iron-coated silicon substrate at $7.0\times {}10^{2}$ $\hbox {s}^{-1}$ and $3.7\times {}10^{3}$ $\hbox {s}^{-1}$. The data collected at $3.7\times {}10^{3}$ $\hbox {s}^{-1}$ are offset by $10^{-1}$ in the vertical axis. The insert compares the two shear rates for each angle, where the data collected at $\theta = 2.3^{\circ }$ are offset by $10^{-1}$ in the vertical axis. The lines between data points in the insert are linear interpolation lines to guide the eye.

The tribometer was loaded with dodecane-$\hbox {d}_{26}$ which was entrained against the substrate at two maximum shear rates of 7.0 $\times$ 10$^{2}$ $\hbox {s}^{-1}$ and 3.7 $\times$ 10$^{3}$ $\hbox {s}^{-1}$ using roller horizontal velocities of $1.1\times {}10^{-3}$ m $\hbox {s}^{-1}$ and $1.8\times {}10^{-3}$ m $\hbox {s}^{-1}$ and surface velocities of $1.4\times {}10^{-1}$ m $\hbox {s}^{-1}$ and $7.2\times {}10^{-1}$ m $\hbox {s}^{-1}$ respectively. The NR profiles are shown in Fig. 4. The critical edge is located at *Q* = 0.0144 Å$^{-1}$ for both profiles, corresponding to approximately 96% solvent deuteration. The total reflection observed at $Q < 0.0144$ Å$^{-1}$ suggests that dodecane-$\hbox {d}_{26}$ completely wets the area illuminated by the neutron beam as any area within the footprint that remained dry during the experiment would contribute a non-zero gradient to the region of total reflection.

When comparing the data collected at 0.7$^{\circ }$ and 2.3$^{\circ }$ there is a clear difference between the first fringe minimum at *Q* $\approx$ 0.04 Å$^{-1}$ at 7.0 $\times$ 10$^{2}$ $\hbox {s}^{-1}$, which is not reproduced at 3.7 $\times$ 10$^{3}$ $\hbox {s}^{-1}$. As the footprint of the beam along the interface is greater than the width of the meniscus at both shear rates, the measured reflectivity is expected to contain a fractional contribution from the interface that is not contained in the meniscus. The total reflectivity, $R_{\mathrm {tot}}$, can be modelled using Eq. (1).

1 $$\begin{aligned} R_{\mathrm {tot}}&= R_{\mathrm {S}}\times {\bar{\gamma }} + R_{\mathrm {NS}}\times (1-{\bar{\gamma }}). \end{aligned}$$

Here, $R_{\mathrm {tot}}$ is the weighted linear combination of reflectivity arising from the region held within the meniscus, $R_{\mathrm {S}}$, and the region held outside of the meniscus, $R_{\mathrm {NS}}$. The weighting factor, ${\bar{\gamma }}$, is calculated as the average of the shear fraction, $\gamma$, which is the ratio of the meniscus width within the footprint to the total footprint of the beam, weighted by the relative intensity of the footprint. This is further detailed in the Supplementary Information.

**Figure 5:**
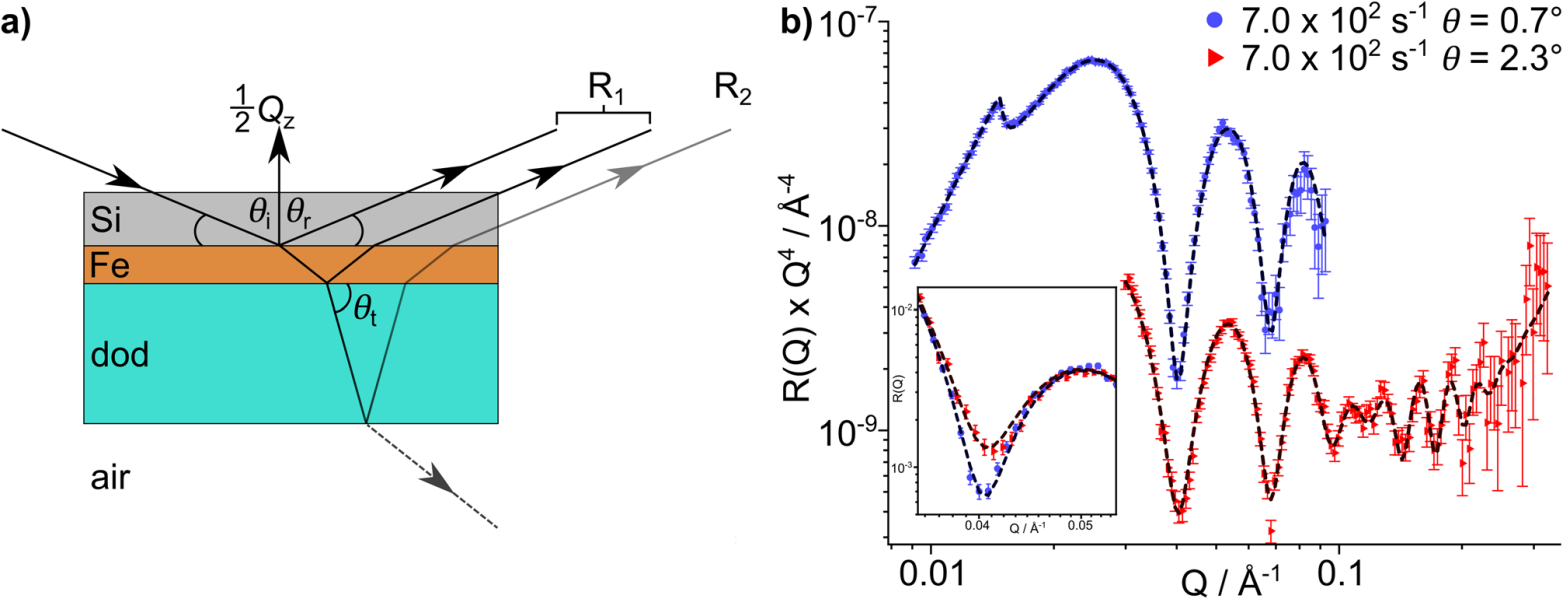
Depiction of reflectivity arising from the non-sheared portion of the interface and the best fit to the NR data collected with dodecane-$\hbox {d}_{26}$ at 7.0 $\times$ 10$^{2}$ $\hbox {s}^{-1}$. (**a**) Schematic of thick film reflectivity. $R_{1}$ is the reflectivity term that accounts for reflection from the immediate Si interface. Neutrons transmitted over the immediate interface will propagate through dodecane at an angle $\theta _{\mathrm {t}}$ before being either reflected or transmitted at the dodecane–air interface. $R_{2}$ is the reflectivity term that accounts for reflection from the dodecane–air interface. (**b**) The best fit, shown by dashed lines, to the NR data collected with dodecane-$\hbox {d}_{26}$ entrained against an iron-coated silicon substrate at 7.0 $\times$ 10$^{2}$ $\hbox {s}^{-1}$. The reflectivity has been modified by a $Q^{4}$ factor to aid comparison. The data collected at $\theta = 2.3^\circ$ is offset by $10^{-1}$ in the vertical axis. The insert shows the unadjusted reflectivity at the first fringe minimum.

Both the $R_{\mathrm {NS}}$ and $R_{\mathrm {S}}$ terms account for reflectivity from the immediate Si interface which includes the sputtered and adsorbed thin films. However, $R_{\mathrm {NS}}$ contains a further reflectivity contribution which results from neutrons propagating over the dodecane film that wets the substrate when not held in the meniscus. Upon reaching the dodecane–air interface, the neutrons are either reflected towards the detector or transmitted into air. Reflection over thicker films on micrometer length scales have been reported previously, and this process is depicted in Fig. 5a^33,34^. The total reflectivity for the non-sheared portion of the interface is given in Eq. (2).

2 $$\begin{aligned} R_{\mathrm {NS}}&= R_1 + \frac{\left( {1-R}_1\right) ^2R_2e^{-\mu L}}{1-R_1R_2e^{-\mu L}}. \end{aligned}$$

Here, $R_1$ is the reflectivity from the immediate Si interface, $R_2$ is the reflectivity from the dodecane–air interface and $\mu$ is the wavelength-dependant neutron attenuation coefficient in dodecane which is shown in the Supporting Information. The neutron path length within the dodecane layer, *L*, is defined as $L = 2d/\sin {\theta _{\mathrm {t}}}$, where $\theta _{\mathrm {t}}$ and *d* are the angle of transmittance and the film thickness of the dodecane layer respectively. Equation (2) accounts for multiple reflections across the thick dodecane film and any resulting attenuation of the neutron beam over this layer. The reflected intensity measured from the sheared portion of the interface, $R_{\mathrm {S}}$, is assumed to arise solely from the immediate interface. It is not expected that significant reflection occurs in the specular direction from the dodecane-PEEK interface due to the angle of reflection adopted when striking the roller. Additionally, the roller has an RMS roughness of 1.5 µm which will diminish the intensity of specular reflection from the roller.

The model defined above was coupled with a conventional multilayer slab model to describe the reflectivity from the thin films present on the substrate surface. Further information on the parameters that describe the layers and the parameter bounds can be found in the Supplementary Information. The best fit to the data collected at $7.0\times {}10^{2}$ $\hbox {s}^{-1}$ is shown by the dashed lines in Fig. 5b. The thickness of the residual dodecane layer which remains on the substrate when not sheared was fit with values of $112^{+85}_{-23}$ µm. The distributions of the layer parameters are shown in Table 1.

**Table 1:** Fitted layer parameters for dodecane-$\hbox {d}_{26}$ entrained against an iron-coated silicon substrate at 7.0 $\times$ 10$^{2}$ $\hbox {s}^{-1}$. The central parameter values are the median values obtained from the bootstrap routine, with the 95% confidence intervals reported in the sub- and superscripts. Those values without uncertainties were held constant. $^{{\text {a}}}$Parameters used to model the sheared layer. $^{{\text {b}}}$Parameters used to model the non-sheared layer.

Layer	$\mathrm {SLD}_{\mathrm {n}}$ / Å$^{-2}\times {}10^{-6}$	$\mathrm {SLD}_{\mathrm {m}}$ / Å$^{-2}\times {}10^{-6}$	Thickness / Å	Roughness / Å
Si	2.07	–	$\infty$	3.0
$\hbox {SiO}_{2}$	3.47	–	$6.4^{+6.5}_{-5.4}$	$4.9^{+1.3}_{-1.4}$
Fe	$7.7^{+0.1}_{-0.1}$	$4.7^{+0.1}_{-0.1}$	$18.6^{+0.2}_{-0.2}{\times 10^{1}}$	$5.3^{+2.9}_{-2.6}$
$\hbox {FeO}_{\mathrm {x}}$	$6.1^{+0.5}_{-0.7}$	$1.3^{+0.0}_{-0.7}$	$32.6^{+4.6}_{-4.5}$	$8.6^{+3.6}_{-4.5}$
Adv. Lay.$^{{\text {a}}}$	$1.4^{+3.9}_{-1.9}$	–	$16.3^{+12.3}_{-12.3}$	$7.5^{+5.5}_{-4.1}$
Adv. Lay.$^{{\text {b}}}$	$1.6^{+3.1}_{-2.2}$	–	$12.8^{+13.2}_{-6.3}$	$7.7^{+5.0}_{-4.1}$

For an acceptable fit, it was necessary to model an additional layer at the iron oxide–dodecane interface, referred to as the ‘Adventitious Layer’. Comparable films have been reported in other NR studies at interfaces with high and low interfacial energies, which are postulated to arise from fluid density depletion and/or gas present at the interface^35–38^. It has also been suggested that impurities within solvents and solutes could form similar layers at solid–liquid interfaces^39,40^. Possible contaminants at the iron oxide–dodecane interface are suggested to be gaseous molecules introduced from the atmosphere or polar contaminants that are native within the solvent, such as ambient dissolved water. Complementary data for the iron oxide–dodecane interface, which was collected using solid–liquid cells under static conditions, was also found to be best represented by the inclusion of an adventitious layer as shown in the Supporting Information. Modelling the data without the adventitious layer resulted in a poorer fit.

To account for possible variation in the adsorbed structure under the two flow environments, separate adventitious layers were modelled for the $R_{\mathrm {S}}$ and $R_{\mathrm {NS}}$ contributions which represent the average surface layer structure whilst sheared and non-sheared. If the adventitious layer is composed of adsorbed gaseous species or water as discussed above, the fitted thicknesses suggest possible multilayering of the adsorbate while static and under shear. Furthermore, if the material adsorbed at the interface is comprised of organic elements such as C, H, N and O, the fitted nuclear scattering length densities, $\mathrm {SLD}_{\mathrm {n}}$, of the adventitious layer indicates that these layers are solvated. An example structure that would fit this description are island-like assemblies of adsorbed material across the interface, with solvent occupying regions between the adsorbate.

The parameter values and uncertainties for the sheared and non-sheared adventitious layers are similar with wide uncertainties. Consequently, the effects of shear on the structure of the adventitious layer are not clear. A possible factor contributing to the wide uncertainties is correlation between layer parameters. Another factor could be the effect of shear itself; for example, the confidence intervals for the sheared layer parameters are marginally wider, which could suggest a greater variation in the structure of the adventitious layer when sheared. However, the fit is less influenced by the parameters of the sheared layer due to the lower weighting of the $R_{\mathrm {S}}$ contribution at 7.0 $\times$ 10$^{2}$ $\hbox {s}^{-1}$. As a result, the parameters of the sheared layer can vary more widely whilst remaining consistent with the data. With this in mind, it would appear that the adventitious layers are equivalent within error.

The fitted magnetic scattering length density, $\mathrm {SLD}_{\mathrm {m}}$, of the iron oxide suggests that the film contains a significant proportion of magnetic iron oxides such as magnetite or maghemite. The fitted $\mathrm {SLD}_{\mathrm {n}}$ for the iron oxide layer is lower than the expected $\mathrm {SLD}_{\mathrm {n}}$ for hematite, magnetite and maghemite at $7.20\times {}10^{-6}$ Å$^{-2}$, $6.95\times {}10^{-6}$ Å$^{-2}$ and $6.67\times {}10^{-6}$ Å$^{-2}$ respectively. The lower $\mathrm {SLD}_{\mathrm {n}}$ could suggest the presence of iron hydroxides which typically have lower atomic densities, and hence lower $\mathrm {SLD}_{\mathrm {n}}$s, than the iron oxides mentioned above^41^. Another possibility is that the iron oxide layer is somewhat porous, with the adventitious layer adsorbing into the pores.

The model used to fit the data collected at $7.0\times {}10^{2}$ $\hbox {s}^{-1}$ does not reproduce the sharp fringe minima in the reflectivity collected at $3.7\times 10^{3}$ $\hbox {s}^{-1}$ as shown in the Supplementary Information. The sharper fringe at $3.7\times 10^{3}$ $\hbox {s}^{-1}$ is thought to result from reduced specular reflection from the dodecane–air interface at higher roller angular velocities. This could be caused by increased attenuation over a thickened wetting dodecane layer and/or from a significant roughening of the interface. The former case has been calculated to require the dodecane layer to thicken substantially, approximately to 400 µm, to produce the sharper fringe minima. While some thickening is expected with greater angular velocities, this scale of thickening would be visible and has not been observed. However, it has been observed that higher roller angular velocities lead to the roughening of the wetting layer, where visible waviness is present for the whole stroke length of the tribometer roller. Images comparing the roughness of the wetting layer at 7.0 $\times$ 10$^{2}$ $\hbox {s}^{-1}$ and 3.7 $\times$ 10$^{3}$ $\hbox {s}^{-1}$ are shown in the Supplementary Information. Therefore, it is suggested that a roughening factor for the dodecane–air interface must be included to suitably model the reflectivity at high roller angular velocities.

#### GMO at the iron oxide–dodecane interface

**Figure 6:**
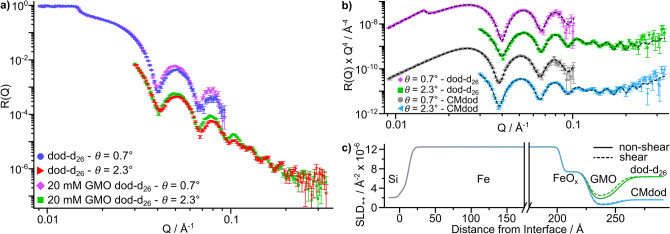
NR data, fits and SLD profiles for the GMO-dodecane systems. (**a**) Comparison of NR data for dodecane-$\hbox {d}_{26}$ with and without GMO entrained against iron-coated silicon substrates at 7.0 $\times$ 10$^{2}$ $\hbox {s}^{-1}$. Data collected at $\theta = 2.3^\circ$ are offset by $10^{-1}$ in the vertical axis. (**b**) NR data for 20 mM GMO in dodecane-$\hbox {d}_{26}$ and CMdod entrained against an iron-coated silicon substrate at 7.0 $\times$ 10$^{2}$ $\hbox {s}^{-1}$. Best fits shown by dashed lines. The reflectivity has been modified by a $Q^{4}$ factor to aid comparison. Data are offset in the vertical axis for clarity. (**c**) Spin-up scattering length density, $\hbox {SLD}_{++}$, profile from the median values of the GMO-dodecane system. A small $\hbox {SiO}_{2}$ layer was modelled between the Si and Fe layer but is not labelled for clarity. Here, $\mathrm {SLD}_{++} = \mathrm {SLD}_{\mathrm {n}} + \mathrm {SLD}_{\mathrm {m}}$.

Two dodecane solutions containing GMO (20 mM) were entrained against an iron-coated silicon substrate at $7.0\times {}10^{2}$ $\hbox {s}^{-1}$ and their NR profiles were collected. The shear rate was achieved using a horizontal velocity of $1.8\times {}10^{-3}$ m $\hbox {s}^{-1}$ and a roller surface velocity of $1.4\times {}10^{-1}$ m $\hbox {s}^{-1}$. One of the GMO solutions was made with 100 % dodecane-$\hbox {d}_{26}$ and the other solution was made with a 29:71 volumetric mixture of dodecane-$\hbox {d}_{26}$:dodecane-$\hbox {h}_{26}$, referred to as CMdod. Figure 6a compares the reflectivity measured at $7.0 \times {} 10^{2}$ $\hbox {s}^{-1}$ with neat dodecane-$\hbox {d}_{26}$ to the reflectivity measured with 20 mM solution of GMO in dodecane-$\hbox {d}_{26}$. While the substrates used for the collection of these profiles are not the same, their reflectivity profiles collected in solvent and air are alike. Therefore, it is expected that the shift in the Kiessig fringes between the datasets is due to the adsorption of GMO at the iron oxide–dodecane interface and not the difference between the two coated substrates.

The two solvent contrasts of the GMO-dodecane system were globally fit using the model described for the dataset collected with neat dodecane, with the adsorbed GMO represented by two layers to account for possible variation with shear. Further details on the model can be found in the Supporting Information. The best fit to the data and the median SLD profile from the parameter distributions are shown in Fig. 6b,c respectively. The parameter distributions from the fit are shown in Table 2. The film thickness of the residual dodecane layer was found to be $104^{+71}_{-33}$ µm. It was found that the data was best described using only one adsorbed layer as models that included separate GMO and adventitious layers resulted in unrealistic parameter values. Therefore, it is expected that the GMO layer will contain some residual adventitious material that was present at the interface before exposure to the GMO solution.

**Table 2:** Fitted layer parameters for the GMO-dodecane solutions entrained against an iron-coated silicon substrate at 7.0 $\times$ 10$^{2}$ $\hbox {s}^{-1}$. The central parameter values are the median values obtained from the bootstrap routine, with the 95% confidence intervals reported in the sub- and superscripts. Those values without uncertainties were held constant. $^{{\text {a}}}$Parameters used to model the sheared layer. $^{{\text {b}}}$Parameters used to model the non-sheared layer.

Layer	$\mathrm {SLD}_{\mathrm {n}}$ / Å$^{-2}$ $\times$ $10^{-6}$	$\mathrm {SLD}_{\mathrm {m}}$ / Å$^{-2}$ $\times$ $10^{-6}$	Thickness / Å	Roughness / Å	Solvation / %
Si	2.07	–	$\infty$	3.0	–
$\hbox {SiO}_{2}$	3.47	–	$12.7^{+2.9}_{-7.4}$	$4.6^{+1.9}_{-3.6}$	–
Fe	$7.9^{+0.1}_{-0.1}$	$4.6^{+0.1}_{-0.1}$	$19.1^{+0.1}_{-0.2}{\times 10^{1}}$	$2.1^{+4.4}_{-1.1}$	–
$\hbox {FeO}_{\mathrm {x}}$	$6.9^{+0.3}_{-0.3}$	$0.5^{+0.3}_{-0.3}$	$23.3^{+0.8}_{-0.8}$	$4.3^{+4.8}_{-2.3}$	–
GMO$^{{\text {a}}}$	0.21	–	$24.3^{+9.9}_{-10.2}$	$6.7^{+3.3}_{-4.7}$	$35.2^{+45.3}_{-35.2}$
GMO$^{{\text {b}}}$	0.21	–	$25.8^{+4.4}_{-5.2}$	$7.7^{+2.3}_{-5.7}$	$24.5^{+21.6}_{-22.4}$

The data were fit with fixed $\mathrm {SLD}_{\mathrm {n}}$s for both GMO layers but both had a solvation parameter to model mixing with the solvent. Similar values of solvation have been reported for oleic acid adsorbed at iron oxide–dodecane interfaces using polarised NR^42^. After mixing, the $\mathrm {SLD}_{\mathrm {n}}$s are $2.4^{+2.8}_{-2.2}\times {}10^{-6}$ Å$^{-2}$ and $1.7^{+1.3}_{-1.4}\times {}10^{-6}$ Å$^{-2}$ for the sheared and non-sheared GMO layers in the dodecane-$\hbox {d}_{26}$ system respectively. While the $\mathrm {SLD}_{\mathrm {n}}$ for the GMO layers after mixing are similar values to the adventitious layers in the neat dodecane-$\hbox {d}_{26}$ system, the thicknesses of the GMO layers are greater than adventitious layers. This indicates that the interfacial layer consists of a greater amount of organic material when GMO is present in solution, supporting the previous suggestion of GMO adsorption at the interface.

Monolayer films have been suggested multiple times for OFMs adsorbed at solid–liquid interfaces, where thicknesses are lower than the extended length of the adsorbate molecule^43–46^. While the lower values of the GMO thickness distributions agree reasonably with the extended length of GMO, which is $\approx 23.8$ Å, a monolayer structure would imply GMO is adsorbed at the interface near the normal angle with negligible conformation defects. These implications appear inconsistent with the solvation values, which suggest significant mixing of solvent in the interfacial layers. The formation of a GMO monolayer at the normal angle could only then occur if there were substantial order between solvent and the adsorbate, which is not expected. For these reasons, coupled with the thickness values that are greater than the extended length of GMO, it is suggested that GMO does not adopt a monolayer structure upon adsorption at $7.0 \times 10^{2}$ $\hbox {s}^{-1}$. Instead, it is postulated that more complex structures are formed at the interface; adsorbed reverse micelles have been reported from molecular dynamic simulations for GMO adsorbed at hematite and mica surfaces in non-aqueous solvents^47–49^.

The uncertainties in the solvation and thickness parameters for the sheared GMO layer are approximately double those for the non-sheared layer. It is expected that a significant factor for the greater parameter variation in the sheared layer is the lower sensitivity as discussed for the pure solvent system. It is also possible that the variation arises from the formation of a range of adsorbed structures when under different shear environments, as the tribometer does not provide a homogeneous sheared environment. The non-sheared layer is expected to relax from the sheared state during the time taken to traverse the interface (> 18 s), although it is not clear if partial or full relaxation occurs.

### X-ray reflectometry

**Figure 7:**
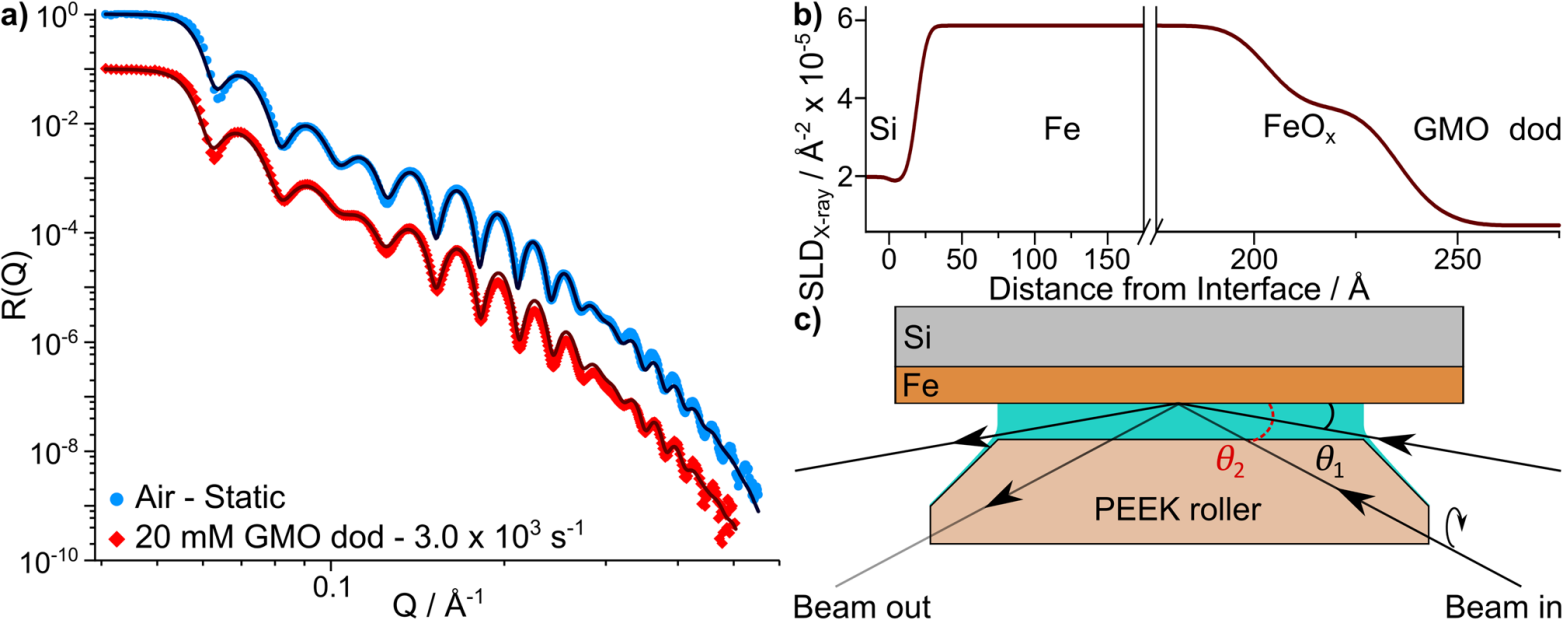
Overview of XRR data and analysis. (**a**) XRR data collected from an iron-coated silicon substrate in air and with a 20 mM GMO dodecane-$\hbox {h}_{26}$ solution entrained against the substrate at 3.0 $\times$ 10$^{3}$ $\hbox {s}^{-1}$. The data collected with the entrained solution has been offset by 10$^{-1}$ in the vertical axis. The solid lines show the fits to the data. Error bars have been removed for clarity. (**b**) The SLD_X-ray_ profile across the interface, constructed using the median parameter values. (**c**) Comparison of the X-ray beam path depending on the scattering angles used. $\theta _{1}$ depicts the beam path at low scattering angles, where the beam propagates through dodecane before and after reflection at the interface. $\theta _{2}$ shows the approximate beam path at greater incident angles, where the X-ray propagates through the PEEK roller and then through dodecane before and after reflection.

An iron-coated silicon substrate was loaded into the tribometer and the XRR profile was measured in air. Afterwards, the tribometer oil bath was loaded with a 20 mM solution of GMO in dodecane-$\hbox {h}_{26}$ and the roller was positioned 200 µm from the substrate surface. The roller was set to a surface velocity of $6.0\times {}10^{-1}$ m $\hbox {s}^{-1}$ whilst held in a horizontal position, resulting in a shear rate of $3.0\times {} 10^{3}$ $\hbox {s}^{-1}$. Subsequently, the XRR profile was collected and is shown along with the air contrast in Fig. 7a. Fits to the data are also shown which were produced by a global fit to both datasets using a multilayer slab model. The model used the same layer properties for the coated Si substrate but the beam-in parameters were chosen to match the different properties of air and dodecane. The fitted layer parameters are shown in Table 3 and the resultant median SLD_X-ray_ profile for the GMO-dodecane system is shown in Fig. 7b.

The model used to fit the dataset collected with GMO includes a transmission scale factor to account for the attenuation of the X-ray beam before and after reflection at the substrate. As the roller is positioned close to the substrate surface, the beam will propagate through dodecane at low $\theta {}$ and through PEEK and dodecane at higher $\theta {}$ as depicted in Fig. 7c. As the beam propagates through both materials, the beam is attenuated resulting in a reduced intensity of coherent scatter falling on the detector. At low $\theta {}$ any angle change upon refraction at the air-dodecane interface is negligible as it is less than 2 $\times {}10^{-7}$ degrees. In the case of a 200 µm gap, the influence of any meniscus curvature is also negligible as the maximum deviation of intensity is less than the measured reflectivity error.

At higher $\theta$ the X-ray beam propagates through the PEEK roller and then through dodecane before and after reflection. The X-ray beam initially strikes the 45$^{\circ }$ chamfered edge of the roller, where the refractive angle follows Snell’s law. The beam is then refracted at the horizontal PEEK-dodecane interface and propagates towards the substrate through dodecane. After reflection, the X-ray beam propagates in the direction of the detector through dodecane and PEEK with a similar path to the incident beam. The intensity at the detector is calculated using the path lengths and the pre-determined linear attenuation coefficients of dodecane and PEEK which are 0.205 $\hbox {cm}^{-1}$ and 0.388 $\hbox {cm}^{-1}$ respectively^50^. The calculation of the path lengths and attenuation factors in dodecane and PEEK is detailed in the Supplementary Information. The transmission scale factor was not included when modelling the data collected with the substrate in air as the roller was not held near the surface and hence did not cross the beam path.

**Table 3:** XRR fitted parameter values for the iron-coated silicon substrate in air and with a 20 mM GMO solution entrained against the substrate at $3.0\times {}10^{3}$ $\hbox {s}^{-1}$. The central parameter values are the median values obtained from the bootstrap routine, with the 95% confidence intervals reported in the sub- and superscripts. Those values without uncertainties were held constant. $^{{\text {a}}}$Layer only included in the 20 mM GMO solution model.

Layer	SLD_X-ray _/ Å$^{-2}\times {}10^{-6}$	Thickness / Å	Roughness / Å
Si	19.8	$\infty$	3.0
$\hbox {SiO}_{2}$	18.6	$19.3^{+10.5}_{-14.1}$	$5.5^{+2.8}_{-0.2}$
Fe	$58.6^{+2.4}_{-1.2}$	$18.4^{+0.1}_{-0.1}{\times 10^{1}}$	$7.2^{+1.9}_{-0.5}$
$\hbox {FeO}_{\mathrm {x}}$	$37.6^{+1.3}_{-1.8}$	$32.2^{+0.8}_{-0.5}$	$7.9^{+0.3}_{-2.8}$
GMO$^{{\text {a}}}$	8.9	$15.3^{+23.0}_{-12.5}$	$4.9^{+4.5}_{-2.5}$

It was necessary to include a $\theta {}$-offset parameter to appropriately model the critical edge in the data collected from the GMO-dodecane system. This parameter was allowed to vary during the fit as the true value of the offset was not known but was expected to be $\approx -0.01^{\circ }$. After fitting the data, it was found that the $\theta {}$-offset was $-9^{+3}_{-2}\times {}10^{-3}$ degrees. As a result the *Q* values in the dodecane-GMO system are expected to be overestimates by approximately $4^{+1}_{-1}\times {}10^{-3}$ Å$^{-1}$. The best fit without this parameter is shown in the Supporting Information, along with further details on the use of the $\theta {}$-offset parameter.

The distribution of the adsorbed GMO thickness is broader with a lower median value compared to the GMO thickness inferred from the NR analysis at $7.0\times {}10^{2}$ $\hbox {s}^{-1}$. It is expected that the poor SLD_X-ray_ contrast between the solvent and the GMO leads to the wide range of GMO thicknesses that are consistent with the data. As a result, a comparison of the GMO structure at $7.0\times {}10^{2}$ $\hbox {s}^{-1}$ and $3.0\times {}10^{3}$ $\hbox {s}^{-1}$ is not appropriate, aside from noting that the fitted thickness of GMO at $7.0\times {}10^{2}$ $\hbox {s}^{-1}$ falls within the range determined at $3.0\times {}10^{3}$ $\hbox {s}^{-1}$. While some variation in the GMO thickness may be expected with the increase in shear rate, it is not expected that the adsorbed layer would be substantially thicker or thinner. Nevertheless, the reasonable fit shows that data collected from an interface under shear with the tribometer can be interpreted.

## Discussion

The use of a novel tribometer designed for the in-situ study of surfactants under shear at solid–liquid interfaces using NR and XRR has been demonstrated by studying the adsorption of GMO at the iron oxide–dodecane interface. NR data was collected with neat dodecane-$\hbox {d}_{26}$ entrained against an iron-coated silicon substrate at two shear rates of 7.0 $\times$ 10$^{2}$ $\hbox {s}^{-1}$ and 3.7 $\times$ 10$^{3}$ $\hbox {s}^{-1}$. It was shown that the NR data collected at the lower shear rate could be modelled by considering the total reflectivity as the weighted sum of the reflection from the sheared and non-sheared portions of the interface. Additionally, the reflectivity from the non-sheared portion of the interface was considered as the combination of reflectivity arising from both the immediate substrate-dodecane interface and the distant dodecane–air interface. NR data collected at the higher shear rate displayed sharper fringe minima that were not reproducible when using this model. It is postulated that higher roller angular velocities increase the roughness of the dodecane–air interface, resulting in reduced specular reflectivity in fringe minima. Therefore, a knowledge of the roughness is essential for modelling the reflectivity at higher shear rates. It would be possible to remove the additional reflectivity from the non-sheared portion by restricting the beam footprint to fall only within the sheared meniscus region. This would have the additional benefit of increasing the sensitivity of the measurement towards the structure of the sheared layer. However, the time required to achieve satisfactory counting error would increase significantly. It would also be possible to increase the size of the meniscus by using a roller with a greater radius, although it is estimated that in order to double the meniscus width, a roller with approximately four times the radius is required. This has the drawback of requiring larger quantities of expensive deuterated material.

It was found when fitting the NR data collected with neat solvent that it was necessary to include an interfacial layer of which the exact nature is unclear. It is suggested that this layer consisted of water or adsorbed atmospheric gases, which may have adsorbed prior to or during the measurement as the tribometer has an open sample environment. It was also shown that GMO adsorbs at the iron oxide–dodecane interface from solution to form an interfacial layer with a thickness of $24.3^{+9.9}_{-10.2}$ Å at 7.0 $\times$ 10$^{2}$ $\hbox {s}^{-1}$ and a thickness of $25.8^{+4.4}_{-5.2}$ Å when not under direct shear. This suggests GMO adsorbs to form a layer that is thicker than a simple monolayer which appears to be insensitive to the effects of shear at 7.0 $\times$ 10$^{2}$ $\hbox {s}^{-1}$. The precise structure of the adsorbed GMO is not known.

XRR data was collected with a 20 mM solution of GMO in dodecane-$\hbox {h}_{26}$ entrained against an iron-coated silicon substrate at $3.0\times {}10^{3}$ $\hbox {s}^{-1}$. A model accounting for attenuation across the beam path before and after reflection was presented and used to fit the sheared XRR data. A GMO thickness of $15.3^{+23.0}_{-12.5}$ Å was found to be consistent with the data, where the wide confidence intervals are thought to result from the poor solvent-adsorbate contrast. Consequently, it is not possible to draw comparisons between the GMO structures at the two different shear rates, and it is difficult to suggest a suitable structure for the interfacial GMO layer at $3.0\times {}10^{3}$ $\hbox {s}^{-1}$. It is suggested that XRR with the tribometer would be a powerful technique for studying surface-active compounds with greater electron densities under shear, such as inorganic friction modifiers.

## Materials and methods

### Materials

Polished silicon blocks with a RMS roughness of 3 Å and dimensions of 55 × 55 × 10 (l × w × h) mm were purchased from Pi-Kem, UK. These were sputter coated with an iron layer by Nano Optics Berlin, Germany, to provide a smooth iron oxide layer. Glycerol monooleate (> 99%) was purchased from Sigma Aldrich and was stored below 0 $^{\circ }$C. *n*-dodecane-$\hbox {h}_{26}$ was purchased from Fisher (> 99% purity, Acros Organics). *n*-dodecane-$\hbox {d}_{26}$ was obtained from Cambridge Isotopes, US (> 98% deuterated, 98% purity).

### Neutron reflectometry

NR experiments with the tribometer were conducted on INTER at ISIS, UK^51^. Neutrons with approximate wavelengths, $\lambda$, of 2–17 Å were selected and the reflectivity profiles were collected at two scattering angles, $\theta$, of 0.7$^{\circ }$ and 2.3$^{\circ }$. This resulted in a *Q* range of 0.009–0.300 Å$^{-1}$, where *Q* is the momentum transfer and is defined as $Q = 4\pi \sin {\theta }/{\lambda }$. The standard deviation $\mathrm {d}Q/Q$ resolution was 2%. The neutron guide slits were set to give a footprint of approximately 40.7 mm along the interface, which can be estimated to have a trapezoid intensity distribution with a 25.4 mm region of homogeneous intensity at the centre.

The iron-coated silicon substrates were cleaned by UV-ozone (20 min) before being mounted in the tribometer. The tribometer roller and oil bath were washed with *n*-dodecane-$\hbox {h}_{26}$ and dried with a $\hbox {N}_{2}$ stream. The tribometer was then loaded with 10 ml of sample solution, and the roller’s angular velocity and horizontal velocity were set at specific values. The temperature of the oil bath and substrate were kept constant at 25 $^{\circ }$C.

The reflection data were normalised with direct beam measurements that were collected with the sample in the beam while using the same slit settings as the reflection measurements. The reduced data were then fit using GenX 2.10 which uses the Parratt recursive formalism to minimise $\chi ^{2}$, the least-squares error between the experimental and simulated reflectivity profiles^52,53^. To model the magnetic domain scattering on a non-polarised instrument, an evenly weighted linear combination of the down- and up-spin reflectivity contributions was used. This approach assumes the size of the magnetic domains in the iron and iron oxide layers are larger than the neutron coherence length. 95% confidence intervals were estimated using a bootstrap resampling routine combined with a differential evolution algorithm (further information in the Supplementary Information).

### X-ray reflectometry

XRR experiments with the tribometer were conducted on I07 at Diamond Light Source, UK^54^. The photon energy was 25.0 keV, corresponding to a wavelength of 0.496 Å. A range of scattering angles, $\theta {} = 0.092^{\circ }$–$1.140^{\circ }$, were used to provide a *Q* range of 0.041–0.504 Å$^{-1}$. The reflected intensity was measured with a Pilatus 100K 2D detector with a count time of one second per scattering angle. The $2\theta {}$ angular resolution was $0.01^{\circ }$, resulting in a momentum transfer standard deviation resolution, $\mathrm {d}Q$, of $2.2\times {}10^{-3}$ Å$^{-1}$. At low $\theta {}$ the sample was over-illuminated by the X-ray beam which had a full width at half maximum vertical height of approximately 100 µm. The temperature of the oil bath and substrate were held at 25 $^{\circ }$C. The same cleaning procedures used in the NR experiments were used to prepare the substrates and the tribometer prior to collection of XRR profiles.

The collected data were reduced with the RodAn package in DAWN, where a region-of-interest with an area of $7\times {}7$ pixels was used to define the specular reflection^55^. Linear background subtraction was performed using two adjacent areas of $5\times 5$ pixels to define the background intensity. The data were also corrected for any over-illumination at low $\theta {}$. The reduced data were then fit using GenX 2.10 by reducing the modulus of the difference between the logarithms of the simulated and real data. Confidence intervals were estimated through the same bootstrap routine as the NR fits.

## Supplementary Information

Supplementary Information.
