# Supplementary material for: Towards a neutron and X-ray reflectometry environment for the study of solid–liquid interfaces under shear
Source: Sci Rep. 2021 May 6;11:9713. doi: 10.1038/s41598-021-89189-1 (PMC8102579; doi:10.1038/s41598-021-89189-1)
Supplement: Supplementary file 1 — Supplementary Information. [file 41598_2021_89189_MOESM1_ESM.pdf]

Towards a neutron and X-ray reflectometry environment for the study of solid-liquid interfaces under shear

SUPPORTING INFORMATION

Alexander J. Armstrong[1], Thomas M. McCoy[1], Rebecca J. L. Welbourn[2], Robert Barker[3], Jonathan L. Rawle[4], Beatrice Cattoz[5], Peter J. Dowding[5], and Alexander F. Routh[1, *]

[1]*BP Institute and Department of Chemical Engineering and Biotechnology, University of Cambridge, UK*

[2]*ISIS Neutron and Muon Source, Didcot, UK*

[3]*School of Physical Sciences, Ingram Building, University of Kent, Canterbury, UK*

[4]*Diamond Light Source Ltd, Diamond House, Harwell Campus, Didcot, OX11 0DE, UK*

[5]*Infineum UK Ltd, Milton Hill, UK*

[*]Correspondence to afr10@cam.ac.uk

March 21, 2021

Neutron reflectometry model details and fit procedure

Model details

For the neutron reflectometry data analysis, the magnetic scattering length density of the iron layer, $\mathrm{SLD_m}$, was fitted as a function of the iron magnetic moment, μ, and the iron nuclear scattering length density, $\mathrm{SLD_n}$, as shown in Equation 1.

$$\mathrm{SLD_m} = \mu \times \frac{\mathrm{SLD_n}}{b} \times C \tag{1}$$

Here, μ has units of Bohr magneton, μ_B, b is the bound coherent scattering length of iron with a value of 9.45×10^{-5} Å and C is a constant of 2.645×10^{-5} Å μ_B^{-1}. This method was not used for fitting the magnetic scattering length density of the iron oxide layer as the precise stoichiometric formula for the iron oxide layer is not known.

The fits to the NR data collected with the GMO/dodecane systems also modelled a solvation for the GMO layers. Here, the scattering length density of GMO, $\mathrm{SLD_n^{GMO}}$, could be adjusted by some fraction, ϕ, of the solvent scattering length density, $\mathrm{SLD_n^{dod}}$, resulting in a solvated layer with a scattering length density of $\mathrm{SLD_n^{solv}}$ as shown in Equation 2.

$$\mathrm{SLD_n^{solv}} = \mathrm{SLD_n^{dod}} \times \phi + \mathrm{SLD_n^{GMO}} \, (1 - \phi) \tag{2}$$

The $\mathrm{SLD_n^{GMO}}$ parameter was held at a fixed value of 0.21×10^{-6} Å^{-2} during the fit procedure.

Fit procedure

The procedure used to generate the best fit and the bootstrap analysis to the NR data is outlined here. The intensity scale factor, I_0, the background parameter, Bkg, and dodecane $\mathrm{SLD_n}$ were fixed at pre-determined values by initially fitting them to truncated parts of the datasets. For the datasets collected at $\theta = 0.7°$ with dodecane-d$_{26}$, I_0 and the dodecane-d$_{26}$ $\mathrm{SLD_n}$ were fit against the first 28 data points. For the datasets collected at $\theta = 0.7°$ that do not have a critical edge, such as the CMdod contrasts, the first 40 data points were chosen to fit I_0 while using a fixed $\mathrm{SLD_n}$ value for the dodecane. For the datasets collected at $\theta = 2.3°$

the intensity scale factor was fit to the first 10 data points. The values of the background parameters for the datasets collected at $\theta = 2.3°$ were fixed by fitting them to the last 15 data points. For each step, the fit procedure was halted when χ^2 reached a steady value for 100 iterations.

The full dataset was then used to infer the parameter values of interest. The initial values of the parameters along with the upper and lower bounds of the parameters that were fit in the NR data are listed in Table S1 and Table S2 for the fits to the solvent and GMO data respectively. The best fit was taken when χ^2 reached a steady value for 100 iterations. The model was then used in conjunction with the bootstrap procedure to estimate the distribution of the parameter values.

Uncertainty analysis

A bootstrap resampling routine was used to estimate the 95 % confidence intervals for the fitted parameters in both the neutron and X-ray reflectometry analysis. Further details on bootstrap techniques are found elsewhere.[1] A python program was used to randomly resample each dataset with replacement, and then fit each resampled dataset with the SciPy differential evolution program using the original model and cost function.[2] This procedure was repeated 500 times for each dataset, resulting in a distribution of parameter values, enabling the median and 95 % confidence interval values to be extracted from the resulting distributions. The 95 % confidence limits were calculated as the difference between the median and the 2.5th/97.5th percentiles of the parameter distributions. A Zenodo repository (DOI: 10.5281/zenodo.4406820) contains the data files and scripts used in this work.

Tribometer shear fraction

The average weighted shear fraction, $\bar{\gamma}$, is the weighting factor in the calculation of R_{tot}. The value of $\bar{\gamma}$ at each individual shear rate can be determined following the procedure outlined in Figure S1. It should be noted that this calculation does not account for the effect of gravity on the neutron beam footprint. Following the theory in section 3.1 of the referenced work[3],

we have calculated the shift in the footprint across the sample plane, where the greatest shift across the sample is approximately 2 mm at $\lambda = 17$ Å. This results in a trapezoid intensity distribution that is translated 2 mm towards the incident beam direction. When this change in footprint is applied in the calculation, the greatest difference in the shear fraction is 1.3 % at a shear rate of 7.0×10^2, where the meniscus width is 11.5 mm. However, the difference quickly falls away to < 1 % by 15 Å, and to even lower values beyond that. Furthermore, at the higher shear rate, with a meniscus width of 20.0 mm, the difference in the shear fraction decreases to 0.8 % (for 17 Å). As such, this effect is deemed negligible and does not need to be included in the calculation. It is suggested that gravitational effects should be taken into consideration when using neutron wavelengths > 20 Å.

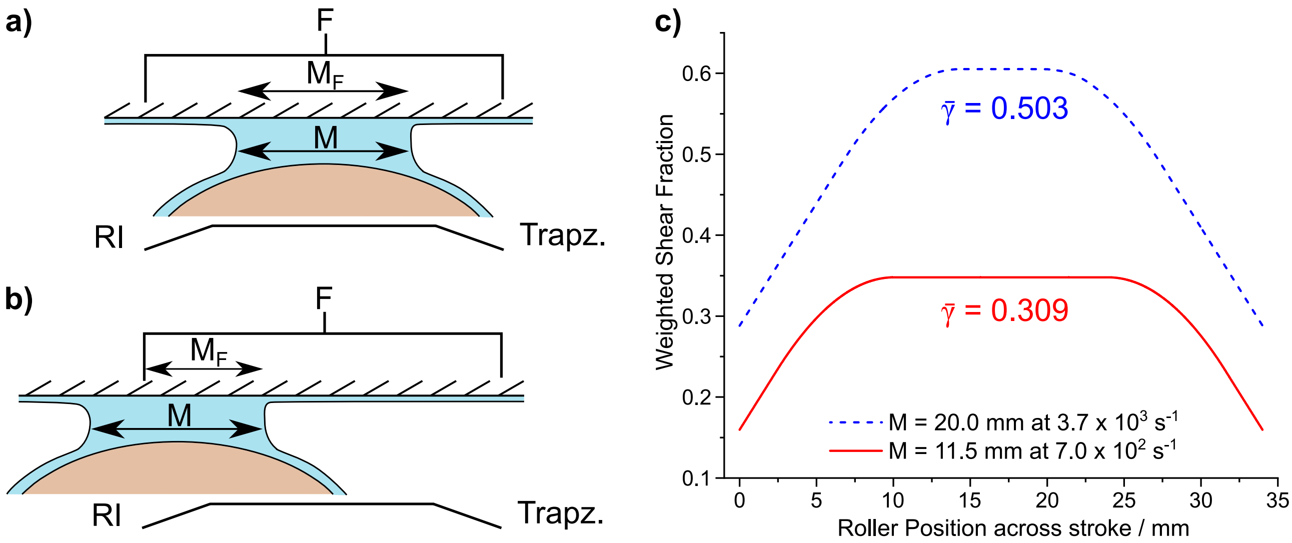

Figure S1. Schematic detailing the determination of the shear fraction for each shear rate. (**a**) Schematic of the roller centred in the middle of the footprint, F. The meniscus width, M, lies completely within F and hence the meniscus width within the footprint, M_F, is equal to M. Below the schematic, the relative intensity (RI) between 0 and 1 is shown by the trapezoid distribution. To calculate the shear fraction at all positions across the footprint, the ratio, M_F/F, must be weighted by the total intensity within F. (**b**) This schematic represents the scenario when the roller has moved some distance across its stroke. Some of the meniscus width lies outside of the footprint, leading to $M_F \neq M$. Therefore, the shear fraction is reduced from its maximum value of M/F. (**c**) The weighted shear fraction, γ, as a function of roller position across the stroke for meniscus widths of 20.0 mm and 11.5 mm. The centre of the roller stroke is 17 mm. As the roller reciprocates across the interface many times during an experiment, the average of the weighted shear fraction, $\bar{\gamma}$, is required. The value of $\bar{\gamma}$ is shown for both shear rates.

Neutron attenuation coefficients

The neutron attenuation coefficients for dodecane are required to model the reflectivity from the immediate sputtered substrate interface and the dodecane-air interface separated by a layer of dodecane with a thickness on the micrometer length scale. The wavelength-dependant neutron attenuation for three volumetric mixtures of dodecane-d_{26}:dodecane-h_{26} (100:0, 29:71 and 0:100) were measured on the ZOOM instrument at ISIS, UK, and are shown in Figure S2. The data were fit with third order polynomials and the coefficients were used in the fitting procedure.

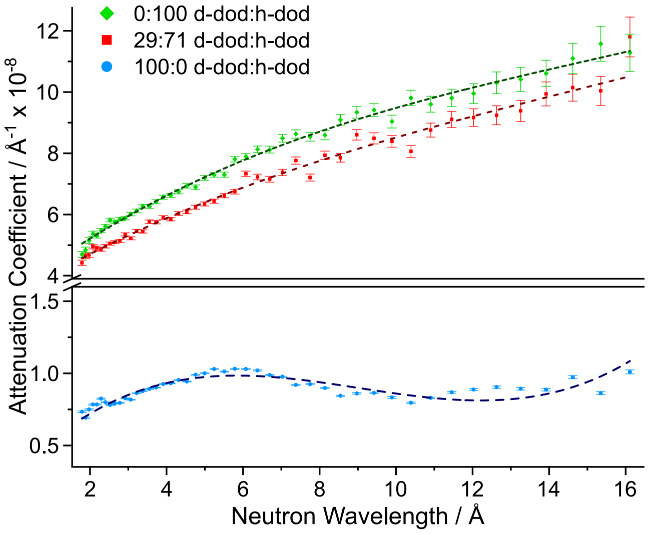

Figure S2. Neutron attenuation coefficients for three dodecane contrasts measured on ZOOM, ISIS, UK. The contrasts are neat dodecane-h_{26}, a 29:71 volumetric mixture of dodecane-d_{26}:dodecane-h_{26}, and neat dodecane-d_{26}.

Iron oxide-dodecane interface when static

NR data of an iron-coated silicon substrate against neat dodecane-d_{26} and dodecane-h_{26} in a PTFE solid-liquid cell under static conditions are presented in Figure S3. This particular substrate was used in the tribometer experiments with the 20 mM GMO-dodecane solutions presented in the main article. The data were modelled with an adventitious layer in the same manner as the tribometer-dodecane experiments and were also modelled as a bare iron oxide-dodecane interface without an adventitious layer. The former model was found to result in a better fit. The parameter values obtained from the bootstrap fitting routine for the model including the adventitious layer are shown in Table S3.

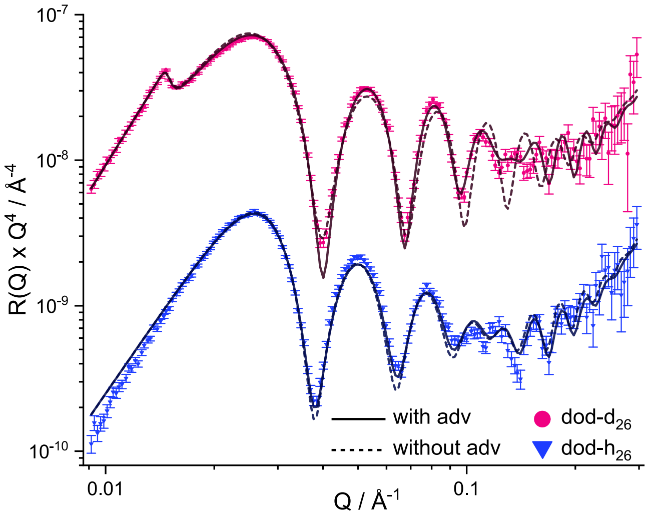

Figure S3. Fits to NR data of dodecane-d_{26} and dodecane-h_{26} against an iron-coated silicon substrate. The data collected with dodecane-h_{26} is offset by 10^{-1} in the vertical direction. The solid line represents the best fit to the data where the model includes an adventitious layer, and the dashed line represents the best fit without inclusion of the adventitious layer.

Modelling the high shear rate data

It is stated in the main article that the model used to fit the NR data collected at 7.0×10^2 s^{-1} does not reproduce the sharp fringe minima in the data collected at 3.7×10^3 s^{-1}. Some exemplar fits using the model outlined in the main article to the data collected at 3.7×10^3 s^{-1} are shown in Figure S4. The fit to data collected at both scattering angles is obtained using an average thickness of 200 μm for the residual dodecane layer that wets the substrate in the non-sheared region. This value represents the upper bound used in the fitting of the 7.0×10^2 s^{-1} data and the minimum roller-substrate gap. The quality of the fit is satisfactory for the data collected at $\theta = 0.7°$. However, a discrepancy between the fit and the data collected at $\theta = 2.3°$ is apparent in the first fringe minima. A comparison of how the fit differs when the thickness of the dodecane layer changes is also shown, where thicker films show an improved fit. It appears that the dodecane thickness has to approach 400 μm to model the sharp fringe minima appropriately; the true thickness of the dodecane layer is not known but it is not expected that the dodecane layer has a thickness on this scale.

Roughening of the wetting dodecane layer

One possible explanation for the sharper fringe minima in the data collected at 3.7×10^3 s^{-1} is roughening of the residual dodecane layer from the greater angular velocities of the roller.

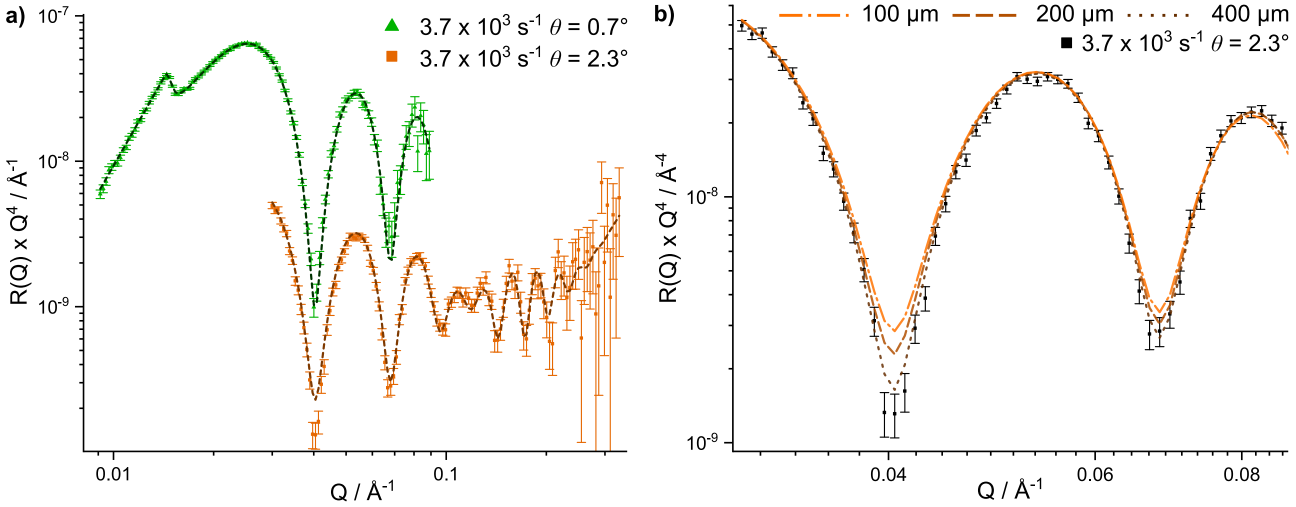

Figure S4. Fits to NR data of dodecane-d_{26} entrained against an iron-coated silicon substrate at 3.7×10^3 s^{-1}. (**a**) Comparison of best fit to the data, modelled with a residual dodecane thickness of 200 µm. The data collected at $\theta = 2.3°$ is offset by 10^{-1} in the vertical axis. The dashed lines show the fit. (**b**) Comparison of three fits to the first two fringe minima of the data collected at $\theta = 2.3°$. The fits used three residual dodecane layer thickness that are listed in the key.

The roughening effect is demonstrated in Figure S5 where three images are presented which show the reflection of a screw on the far-side of the tribometer from the mirrored surface of the substrate. One of the images has been captured with a dry substrate without the roller moving, while the other images have been captured with entrained dodecane on the substrate surface using roller surface velocities of 1.4×10^{-1} m s^{-1} and 7.2×10^{-1} m s^{-1} and a roller horizontal velocity of 1.8×10^{-3} m s^{-1}. These velocities correspond to maximum shear rates of 7.0×10^2 s^{-1} and 3.7×10^3 s^{-1}. When the substrate is dry, an undistorted and focused image is formed. When entraining dodecane onto the substrate the reflection must propagate through the wetting layer of dodecane before reaching the camera. If the surface of the wetting layer is not mirror-like, distortions will be apparent in the captured image.

There is little difference between the images captured with the dry substrate and with the dodecane entrained at 7.0×10^2 s^{-1}, suggesting that the dodecane-air interface is reasonably smooth. However, in comparison to the former two images, the image captured at 3.7×10^3 s^{-1} shows significant distortion. The texture of the dodecane-air interface has been visualised by focussing the camera onto the substrate, where substantial waviness is visible across the entire substrate.

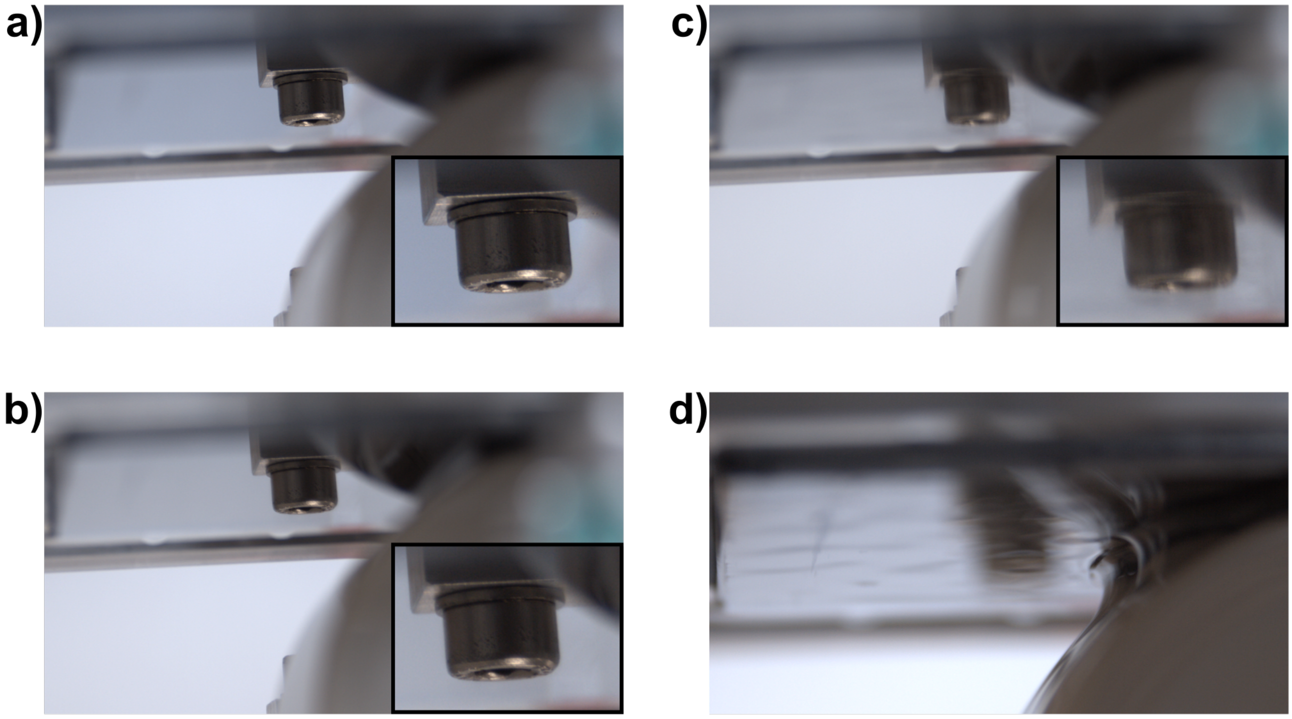

Figure S5. Comparison of the reflection from a Si substrate at increasing angular velocities. (**a**) Image captured with a dry substrate. The screw shown in the reflection from the substrate is the focus point. Insert is a scaled view of the screw. (**b**) Image captured with the substrate wetted with dodecane, where the dodecane is entrained onto the surface at 7.0×10^2 s^{-1}. (**c**) Image captured with the substrate wetted with dodecane. Dodecane entrained onto the surface at 3.7×10^3 s^{-1}. (**d**) Image captured at 3.7×10^3 s^{-1}, but with focus shifted to the substrate. Clear undulations are seen on the dodecane surface across the substrate.

X-ray reflectometry model details and fit procedure

Model details

It was found that it was necessary to include a θ-offset in the model to provide an adequate fit to the XRR data collected under shear with the 20 mM GMO-dodecane system. The best fit achieved without the θ-offset is shown in Figure S6a and the parameter distributions from the bootstrap analysis are shown in Table S4. The majority of the parameter distributions are similar to the parameters inferred from the model including the θ-offset. However, the inferred distribution of the GMO thickness has a greater median value when compared to the distribution inferred using the θ-offset. This skew to higher values is suggested to arise from the poorer fit.

By inspecting the alignment scans conducted before the reflection measurements, as shown in Figure S6b, it is possible to justify the use of the θ-offset parameter as used in the main article. The alignment scans shown involved rocking the sample along the path of the beam to find the angle at which the sample lay flat with respect to the beam. Ideally the scan should result in a sharp symmetric peak as seen in the scan conducted before the reflection measurement in air. The center of the peak can then be chosen as the sample angle to use in the reflection measurements. However, in the case of the alignment scan before the measurement with the entrained GMO-dodecane solution, a more poorly defined peak was found. As the peak and peak maximum are asymmetric about the centre of the scan, the center-of-mass of the peak is likely to be at a negative angle relative to the original angle before the start of the scan procedure. It is suggested that the center-of-mass is approximately $-0.01°$ from the angle at the center of the scan. However, the sample angle used in the reflection experiment with the GMO-dodecane solution was the central angle covered in the scan. Therefore, the calculated θ value for each angle is most likely to be an overestimate of the true angle, and hence by including the θ-offset parameter the overestimated angle can be accounted for.

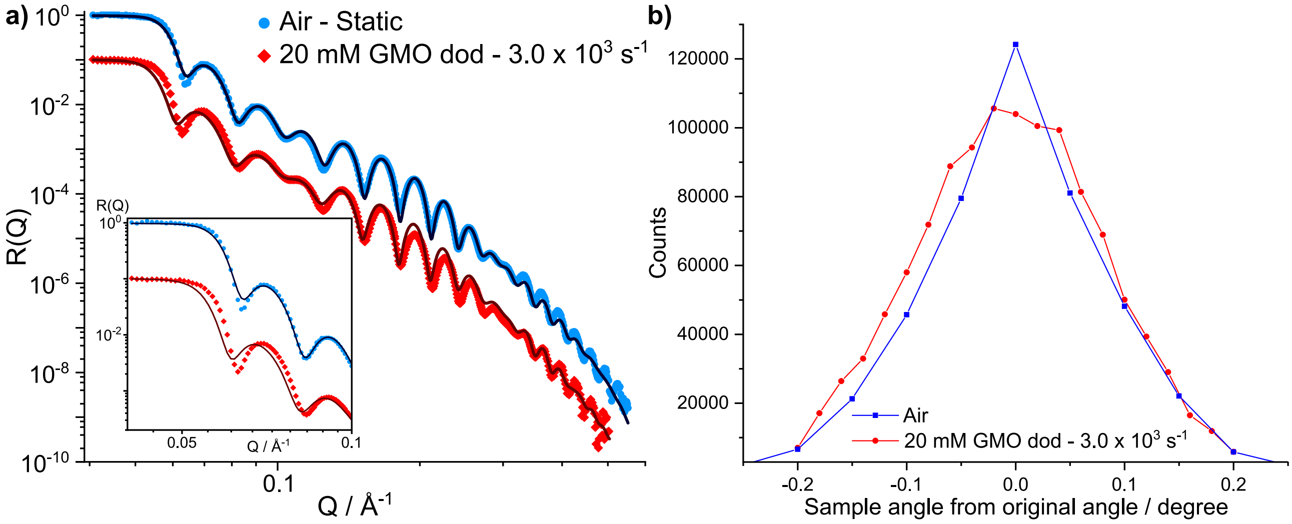

Figure S6. Best fit without a θ-offset included in model and sample angle alignment scans. (**a**) The best fit to the XRR collected with the 20 mM GMO-dodecane solution at 3.0×10^{-3} s^{-1} without including a θ-offset. The insert shows the reflectivity around the critical edge, where a clear offset is shown for the GMO-dodecane contrast. (**b**) Alignment scans for the sample angle which defines the true θ-offset. These scans were conducted statically before the reflection measurements were taken. For the air scan, the roller was not near to the surface, while for the scan before the GMO-dodecane system, the roller was positioned 200 µm from the substrate surface.

Fit procedure

The procedure for obtaining the best fit to the XRR data as shown in the main article is outlined here. The intensity scale factors, I_0, for the air and tribometer contrasts were fixed at pre-determined values by initially fitting them to the first 30 data points of each dataset. The fixed values for the intensity scale factors were then taken after reaching a steady figure-of-merit value for 100 consecutive iterations.

The full dataset was then used to infer the other parameter values of interest. The initial values of the parameters along with the upper and lower bounds of the parameters that were fit in the XRR data are listed in Table S5. The best fit was taken after the figure-of-merit was steady after 100 consecutive iterations. The model was then used in conjunction with the bootstrap procedure to estimate the distribution of the parameter values.

X-ray reflectometry attenuation

The X-ray beam is attenuated as it propagates through the dodecane meniscus and the PEEK roller before and after reflection at the substrate. The extent of the attenuation is determined by the total path length taken in the two materials, which is itself determined by the incident angle and the distance from the center of the beam. As the X-ray beam has a full-width at half-maximum thickness of approximately 100 µm, a proportion of the beam can pass through the PEEK roller while the center of the beam propagates through the dodecane and vice versa. The determination of the path length at high and low theta is described first, followed by the method used to define the attenuation factor at each Q value used in the main article.

Determination of path lengths in dodecane and the PEEK roller

The path followed by the center of the beam at low θ values is depicted in Figure S7. Here, the X-ray beam strikes the air-dodecane interface at a vertical distance from the substrate surface, h_{beam}, which can be calculated using Equation 3, where l is the width of the upper surface of the roller. In the case of the tribometer, l = 40.4 mm.

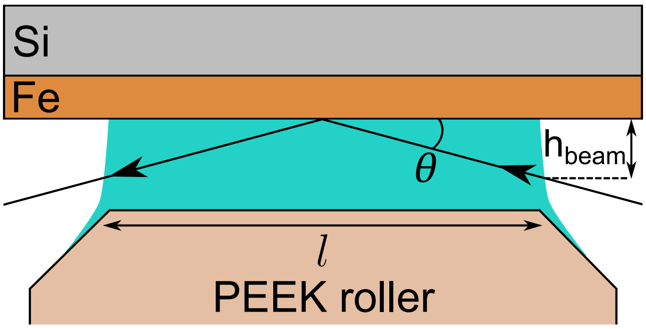

Figure S7. X-ray beam path at low scattering angles. Not drawn to scale.

$$h_{\mathrm{beam}} = \frac{l}{2}\tan\theta \tag{3}$$

The X-ray beam then propagates through dodecane which attenuates the beam before being reflected at the substrate-dodecane interface towards the detector. The attenuation is dependant on the path length within the dodecane, P_{dod}, which can be determined using Equation 4.

$$P_{\mathrm{dod}} = 2\frac{h_{\mathrm{beam}}}{\sin\theta} \tag{4}$$

As stated in the main article, the change in angle is less than 2×10^{-7} degrees when refracted at the air-dodecane interface. Therefore, the path length before and after reflection are considered to be equivalent.

The X-ray beam propagates through the PEEK roller at greater θ values as shown in Figure S8a. This process occurs when h_{beam} is greater than the roller-substrate gap, h_{gap}. The X-ray beam will strike the air-PEEK interface at the chamfered edge of the roller at a vertical distance from the substrate, h_{PEEK}. This can be related to h_{beam} and an additional height contribution from the chamfered edge, h_{edge}, as shown in Equation 5. In the case of the experiment presented in the main article, the X-ray beam does not strike the roller at the flat surface below the chamfered edge as this would only occur at $\theta > 2.72°$.

$$h_{\text{PEEK}} = h_{\text{beam}} + h_{\text{edge}} \tag{5}$$

As shown in Figure S8b, h_{edge}, can be calculated as $h_{\text{edge}} = c_1 \sin\theta_{\text{air}}$, where c_1 is the longest side of the scalene triangle formed with sides a_1 and b_1. The law of cosines can be used to calculate c_1 using a_1, b_1 and α_1, which are given as:

$$a_1 = \tan(45°) \times \ (h_{\text{beam}} - h_{\text{gap}})$$

$$\alpha_1 = 45° - \sin\theta_{\text{air}} \tag{6}$$

$$b_1 = a_1 \frac{\sin\theta_{\text{air}}}{\sin\alpha_1}$$

Following the determination of h_{PEEK} the path length of the incident beam within the PEEK, $P_{\text{in-PEEK}}$, can be calculated following Equation 7. This is depicted in Figure S8c.

$$P_{\text{in-PEEK}} = \frac{h_{\text{PEEK}} - h_{\text{gap}}}{\sin\theta_{\text{PEEK}}} \tag{7}$$

Here, $\sin\theta_{\text{PEEK}}$ is the angle adopted upon refraction at the air-PEEK interface which can be calculated through Snell's Law. Similarly, the angle adopted upon refraction at the PEEK-

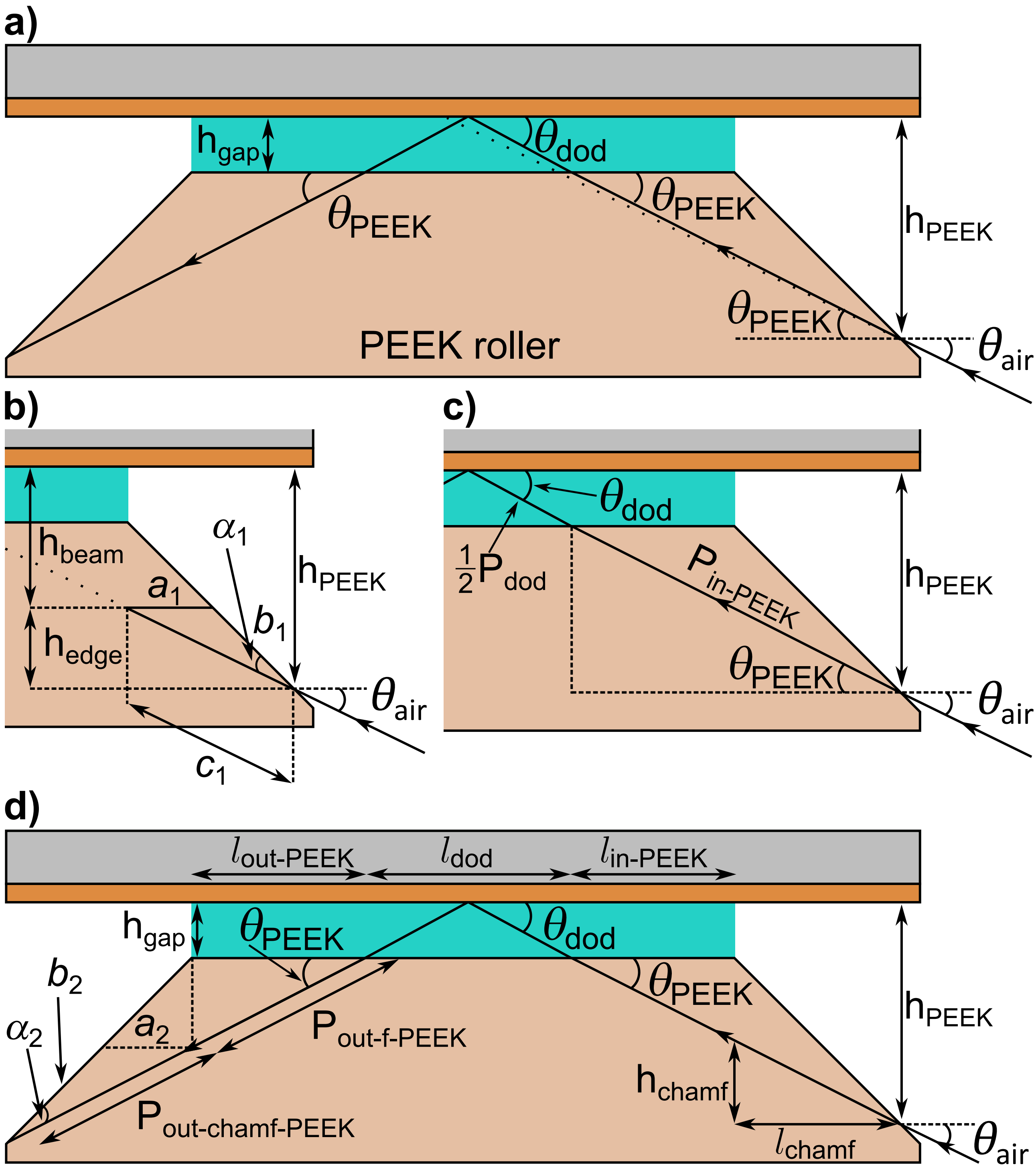

Figure S8. Schematic showing the X-ray beam path through the PEEK roller and the variables used to calculate the total path length. (**a**) X-ray beam propagates through PEEK and dodecane before and after reflection at the substrate. The non-dashed ray shows approximate beam path. (**b**) Zoomed-in perspective of the chamfered edge, where h_{edge} and length c_1 are depicted. (**c**) The path lengths $P_{\text{in-PEEK}}$ and P_{dod} can be calculated with θ_{PEEK} and θ_{dod}. (**d**) Distances used to calculate the path length of the X-ray beam in PEEK after reflection.

dodecane interface, $\sin\theta_{\text{dod}}$, can be calculated via the same principle. The refracted angle θ_{dod} is the final angle at which the beam will impinge on the surface. The total path length in dodecane, P_{dod}, before and after reflection is defined as:

$$P_{\text{dod}} = 2\frac{h_{\text{gap}}}{\sin\theta_{\text{dod}}} \tag{8}$$

The path length through the PEEK after reflection can be calculated by considering the horizontal lengths from the flat upper surface of the roller as shown in Figure S8d. The horizontal length $l_{\text{out-PEEK}}$ can be calculated as:

$$l_{\text{out-PEEK}} = l - (l_{\text{in-PEEK}} + l_{\text{dod}}) \tag{9}$$

where:

$$l_{\text{dod}} = 2\frac{h_{\text{gap}}}{\tan\theta_{\text{dod}}} \tag{10}$$

$$l_{\text{in-PEEK}} = \frac{h_{\text{PEEK}} - h_{\text{chamf}}}{\tan\theta_{\text{PEEK}}} \tag{11}$$

and $h_{\text{chamf}} = \tan\theta_{\text{PEEK}} \times l_{\text{chamf}}$. The term l_{chamf} is given by $l_{\text{chamf}} = \sqrt{c_1^2 - h_{\text{edge}}^2}$. Finally $P_{\text{out-PEEK}}$ can be calculated as shown in Equation 12.

$$P_{\text{out-f-PEEK}} = \frac{l_{\text{out-PEEK}}}{\cos\theta_{\text{PEEK}}} \tag{12}$$

The small remaining path length in the chamfer of the roller, $P_{\text{out-chamf-PEEK}}$, can be calculated using the law of cosines using the variables a_2, b_2 and α_2 that are given as:

$$a_2 = \tan(45°) \times \sqrt{P_{\text{out-f-PEEK}}^2 - l_{\text{out-PEEK}}^2}$$

$$\alpha_2 = 45° - \sin\theta_{\text{PEEK}} \tag{13}$$

$$b_2 = a_2\frac{\sin\theta_{\text{PEEK}}}{\sin\alpha_2}$$

The total path length is then given by the sum of $P_{\text{in-PEEK}}$, P_{dod}, $P_{\text{out-f-PEEK}}$ and $P_{\text{out-chamf-PEEK}}$.

Determination of the X-ray attenuation scale factor

The incident beam has a Gaussian distribution, $G(x)$ with a full-width at half-maximum width of 100 µm. In this work, the full width of the beam has been estimated at ±3 standard deviations (σ). At $\theta \leq 0.240°$ ($Q \leq 0.106$ Å^{-1}) the footprint of the beam is greater than the length of the substrate, l_s, at 55 mm. Therefore, the width of the beam that falls on the substrate is given by $x_\text{w} = \sin\theta \times l_\text{s}/2$. At these low angles, all of the beam that falls on the substrate propagates through the dodecane meniscus, as depicted in Figure S9. The path length within

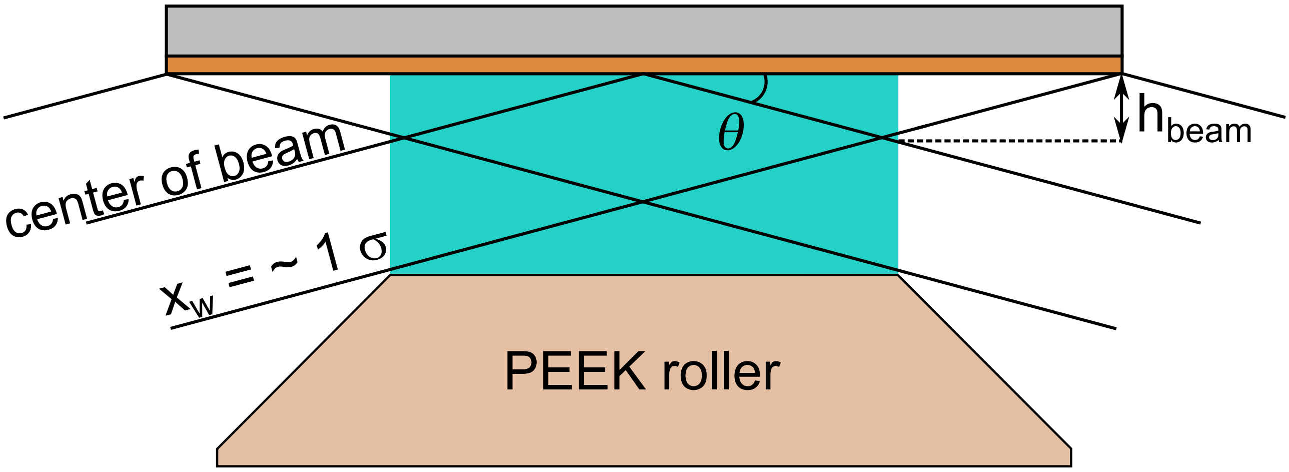

Figure S9. Schematic of the X-ray beam footprint on the substrate at $\theta = 0.092°$. The width of the beam that falls on the substrate is approximately ±1 σ from the beam center. Not drawn to scale.

the dodecane meniscus is given by Equation 4 and is equal for all distances from the center of the beam that fall onto the substrate. The total intensity of the beam that reflects from the substrate is shown in Equation 14.

$$I_\text{s} = \int_{-x_\text{w}}^{+x_\text{w}} G(x)\,\mathrm{d}x \tag{14}$$

The data reduction carried out in DAWN corrected for the footprint effect, and hence I_s can be normalised by itself to obtain a normalised intensity, I_n. The total transmitted intensity, I_tot, is then given by:

$$I_\text{tot} = I_\text{n} e^{-\mu_\text{dod} P_\text{dod}} \tag{15}$$

where μ_{dod} is the X-ray attenuation coefficient in dodecane. While Figure S9 shows a dry area either side of the dodecane meniscus, in reality these areas are coated in dodecane but are not contained within the meniscus. As an approximation, it is assumed that no attenuation via dodecane occurs outside of the meniscus. It is further assumed that any reflection in this area is equivalent to the reflection in the meniscus. The full width of the beam falls within the meniscus at $\theta \geq 0.362°$.

In the range $0.242° \leq \theta \leq 0.566°$ ($0.107 \leq Q \leq 0.250$ Å^{-1}) the outer portion of the beam that hits the substrate propagates through the PEEK roller either before or after reflection while a portion of the beam propagates purely through dodecane. The path length adopted by the beam that propagates purely through dodecane is given by Equation 4. For a given

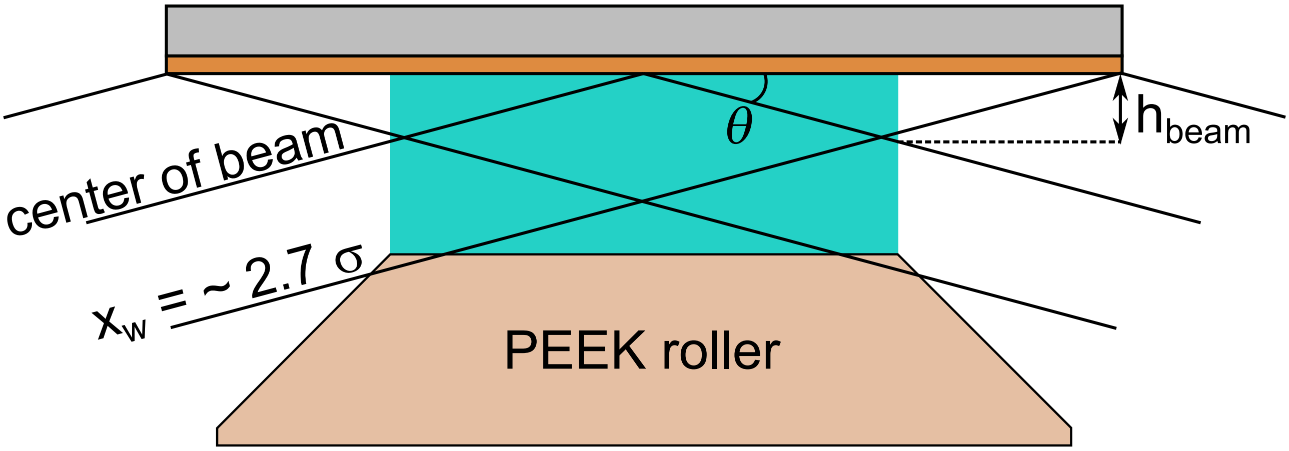

Figure S10. Schematic of the X-ray beam footprint on the substrate at $\theta = 0.242°$. The width of the beam that falls on the substrate is approximately $\pm 2.7\ \sigma$ from the beam center. When $\theta > 0.262°$ the full width of the beam falls on the substrate. Not drawn to scale.

angle, the path lengths in PEEK and dodecane for the outer portion of the beam are a function of the distance from the center of the beam. For a given distance from the center of the beam, x, the path length in PEEK can be determined by the method presented in the previous section. The path length in dodecane can be determined by considering the remaining meniscus width after transmission through the PEEK roller and the angle adopted when refracted at the PEEK-dodecane interface. The transmitted intensity through PEEK can be calculated following Equation 4 but using the path length in PEEK and the attenuation coefficient for PEEK instead of those for dodecane. The total transmitted intensity is then the sum of the attenuated normalised intensities from the outer portion of the beam and the central part of

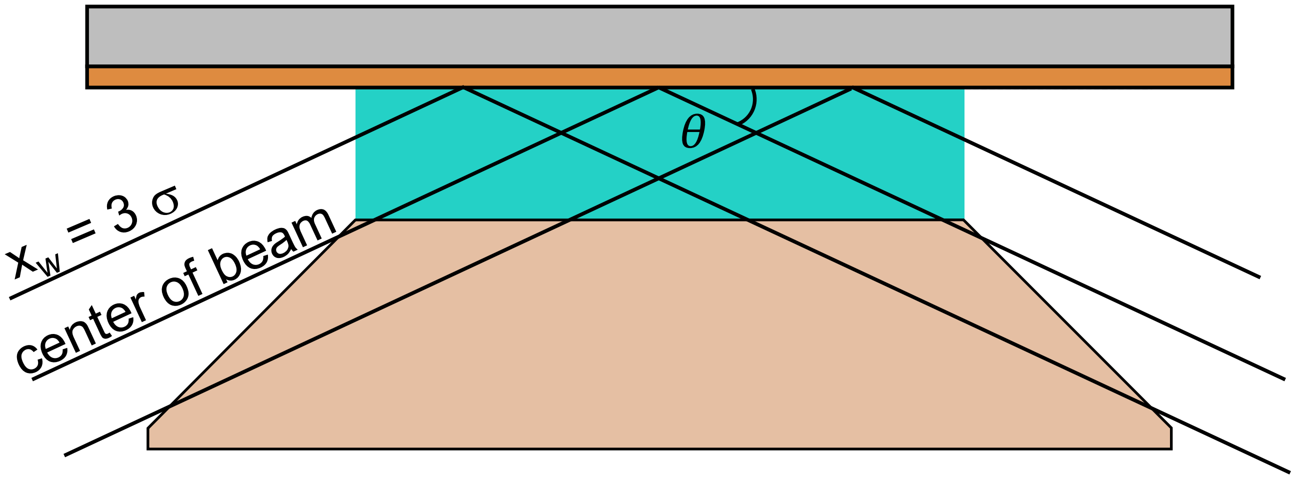

Figure S11. Schematic of the X-ray beam footprint on the substrate at $\theta = 0.568°$. The width of the beam that falls on the meniscus of the substrate is now $\pm 3\ \sigma$. Not drawn to scale.

the beam.

In the range $0.568° \leq \theta \leq 0.928°$ ($0.251 \leq Q \leq 0.410$ Å^{-1}) the central part of the beam passes through the PEEK substrate and then through dodecane before reflection at the substrate as shown in Figure S11. Following reflection the beam then propagates back through dodecane and PEEK towards the detector. This portion of the beam is small at $\theta = 0.568°$ and is almost all of the gaussian beam by $\theta = 0.928°$. The outer portion of the beam propagates through the PEEK roller either before or after reflection. The total intensity in this range is given in the same way in the range $0.242° \leq \theta \leq 0.566°$.

Finally, in the range $0.932° \leq \theta \leq 1.140°$ ($0.412 \leq Q \leq 0.504$ Å^{-1}) all of the beam propagates through the PEEK roller and dodecane before and after reflection. The total intensity in this range is then the attenuated normalised intensity following this path. Figure S12a shows the scale factor calculated as a function of Q. As the data reduction in DAWN normalises the data to the reflection at below the critical edge, the scale factor should be normalised by the transmission at the lowest Q where the beam only passes through dodecane. Therefore, the transmission scale factor is approximately one at low Q and decreases as a larger fraction of the beam propagates through the PEEK roller as Q increases. The gradient of the transmission scale factor becomes less negative at $Q \approx 0.35$ Å^{-1}. This is because the gradient of the average path length in PEEK, as followed by the portion of the beam that propagates through

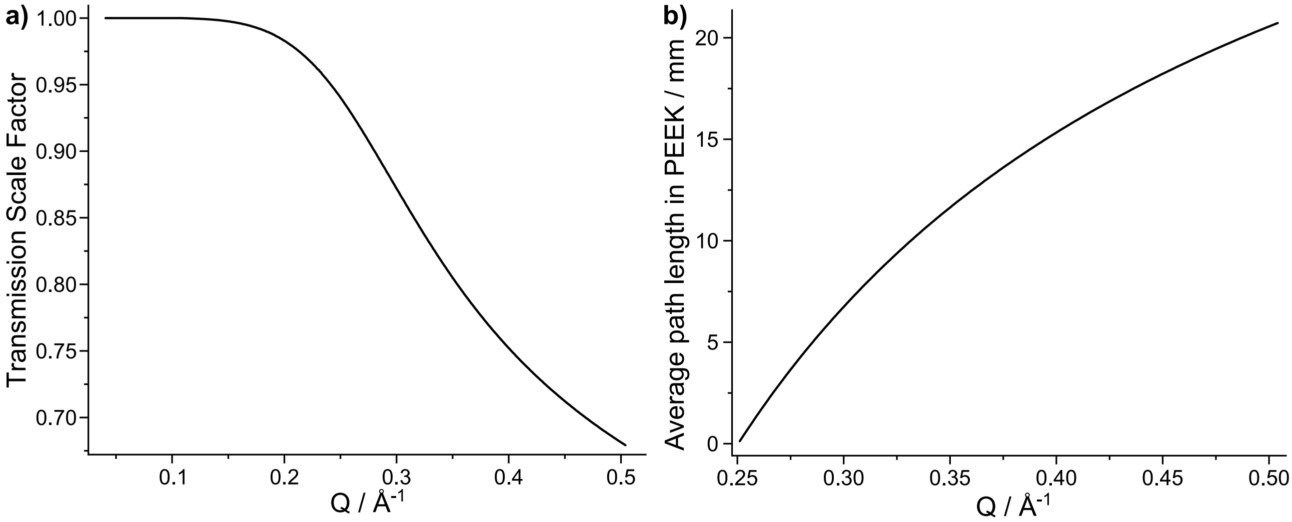

Figure S12. (**a**) The transmission scale factor calculated for each Q point in the X-ray reflectivity data for the 20 mM GMO-dodecane system at 3.0×103 s^{-1}. (**b**) The average path length in PEEK of the portion of the beam that propagates through PEEK and dodecane before and after reflection as a function of Q.

PEEK and dodecane both before and after reflection, as a function of Q decreases as shown in Figure S12b. The modelled reflection in GenX was multiplied by the scale factor at each point in Q in order to account for the attenuation.

Reflection at the PEEK-dodecane interface for the incident and reflected beam has also been considered. In the limiting case of a perfectly flat interface, the maximum reflection is estimated to be 2×10^{-5}. Therefore, to a reasonable approximation, the reflection from the PEEK-dodecane interface can be treated as negligible. It should be noted that the roller's roughness will reduce the transmitted intensity in the specular direction and increase the off-specular intensity.

References

1. Efron, B., & Tibshirani, R. J. *An introduction to the bootstrap* (CRC press, 1994).
2. Virtanen, P. *et al.* SciPy 1.0: Fundamental Algorithms for Scientific Computing in Python. *Nature Methods* **17,** 261–272 (2020).
3. Gutfreund, P. *et al.* Towards generalized data reduction on a chopper-based time-of-flight neutron reflectometer. *J. Appl. Cryst.* **51,** 606–615 (2018).

Table S1. Initial parameter values and the bounds when fitting to the NR data collected with neat dodecane-d_{26} in the tribometer. * – parameters used to model the sheared layer. † – parameters used to model the non-sheared layer.

Layer	Parameter	Initial Value	Bounds	Units
Si	Thickness	∞	-	Å
	Roughness	3	-	Å
	SLD_n	2.07	-	10^{-6} Å^{-2}
SiO_2	Thickness	10	(1, 20)	Å
	Roughness	5	(2, 15)	Å
	SLD_n	3.47	-	10^{-6} Å^{-2}
Fe	Thickness	190	(143, 238)	Å
	Roughness	6	(1, 15)	Å
	SLD_n	8.0	(7.3, 8.1)	10^{-6} Å^{-2}
	μ	2.1	(1.0, 2.2)	μ_B
FeO_x	Thickness	30	(20, 45)	Å
	Roughness	7	(2, 15)	Å
	SLD_n	7.0	(5, 7.2)	10^{-6} Å^{-2}
	SLD_m	0.0	(0, 1.3)	10^{-6} Å^{-2}
Adv*	Thickness	10	(1, 35)	Å
	Roughness	7	(2, 15)	Å
	SLD_n	0.0	(-0.54, 5.5)	10^{-6} Å^{-2}
Adv†	Thickness	10	(1, 35)	Å
	Roughness	7	(2, 15)	Å
	SLD_n	0.0	(-0.54, 5.5)	10^{-6} Å^{-2}
dodecane-d_{26}	SLD_n	6.42	-	10^{-6} Å^{-2}
Misc	Shear fraction	0.309	-	-
	Dodecane thickness	100	(30, 200)	µm
	$I_0, \theta = 0.7°$	0.934	-	-
	$I_0, \theta = 2.3°$	0.981	-	-
	Bkg, $\theta = 2.3°$	2.69	-	10^{-6}

Table S2. Initial parameter values and the bounds when fitting to the NR data collected with the GMO/dodecane solutions in the tribometer. * – parameters used to model the sheared layer. † – parameters used to model the non-sheared layer. ‡ - parameters used to fit data collected with dodecane-d_{26}. ♦ - parameters used to fit data collected with CMdod.

Layer	Parameter	Initial Value	Bounds	Units
Si	Thickness	∞	-	Å
	Roughness	3	-	Å
	SLD_n	2.07	-	10^{-6} Å^{-2}
SiO_2	Thickness	10	(5, 25)	Å
	Roughness	5	(1, 10)	Å
	SLD_n	3.47	-	10^{-6} Å^{-2}
Fe	Thickness	190	(143, 238)	Å
	Roughness	6	(1, 10)	Å
	SLD_n	8.0	(7.1, 8.0)	10^{-6} Å^{-2}
	μ	2.1	(1.6, 2.2)	μ_B
FeO_x	Thickness	30	(20, 40)	Å
	Roughness	7	(2, 15)	Å
	SLD_n	7.0	(5.5, 7.2)	10^{-6} Å^{-2}
	SLD_m	0.0	(0, 1.3)	10^{-6} Å^{-2}
GMO*	Thickness	20	(1, 40)	Å
	Roughness	7	(2, 10)	Å
	SLD_n	0.21	-	10^{-6} Å^{-2}
	Solvation	0	(0, 1)	-
GMO†	Thickness	20	(1, 40)	Å
	Roughness	7	(2, 10)	Å
	SLD_n	0.21	-	10^{-6} Å^{-2}
	Solvation	0	(0, 1)	-
dodecane-d_{26}	SLD_n	6.35	-	10^{-6} Å^{-2}
CMdod	SLD_n	1.61	-	10^{-6} Å^{-2}
Misc	Shear fraction	0.309	-	-
	Dodecane thickness	100	(30, 200)	µm
	$I_0, \theta = 0.7°$ ‡	0.955	-	-
	$I_0, \theta = 2.3°$ ‡	0.910	-	-
	$I_0, \theta = 0.7°$ ♦	0.928	-	-
	$I_0, \theta = 2.3°$ ♦	0.830	-	-
	Bkg, $\theta = 2.3°$ ‡	1.00	-	10^{-6}
	Bkg, $\theta = 2.3°$ ♦	3.04	-	10^{-6}

Table S3. Fitted layer parameters for dodecane-d_{26} and dodecane-h_{26} against an iron-coated silicon substrate under static conditions. The central parameter values are the median values obtained from the bootstrap routine, with the 95 % confidence intervals reported in the sub- and superscripts. Those values without uncertainties were held constant. * - SLD parameter used to model the adventitious layer in dodecane-d_{26}. † - SLD parameter used to model the adventitious layer in dodecane-h_{26}.

Layer	SLD_n / $Å^{-2} \times 10^{-6}$	SLD_m / $Å^{-2} \times 10^{-6}$	Thickness / Å	Roughness / Å
Si	2.07	-	∞	3.0
SiO_2	3.47	-	$5.0^{+2.7}_{-0.0}$	$4.6^{+1.1}_{-1.0}$
Fe	$8.0^{+0.0}_{-0.0}$	$4.9^{+0.0}_{-0.0}$	$19.3^{+0.1}_{-0.1}\times10^{1}$	$4.9^{+1.2}_{-1.2}$
FeO_x	$7.2^{+0.0}_{-0.0}$	$0.0^{+0.0}_{-0.0}$	$26.0^{+1.1}_{-1.3}$	$7.3^{+0.8}_{-1.0}$
Adv. Lay.	$0.1^{+3.4*}_{-0.6}$ $-0.6^{+0.0\dagger}_{-0.0}$	-	$7.8^{+7.7}_{-2.0}$	$11.4^{+2.3}_{-3.9}$

Table S4. XRR fitted parameter values for the iron-coated silicon substrate in air and with a 20 mM GMO solution entrained against the substrate at 3.0×10^3 s^{-1} without the use of a θ-offset. The central parameter values are the median values obtained from the bootstrap routine, with the 95 % confidence intervals reported in the sub- and superscripts. Those values without uncertainties were held constant. * – layer only included in the 20 mM GMO solution model.

Layer	$SLD_{X\text{-ray}}$ / $Å^{-2} \times 10^{-6}$	Thickness / Å	Roughness / Å
Si	19.8	∞	3.0
SiO_2	18.6	$19.2^{+9.3}_{-13.9}$	$5.4^{+3.0}_{-0.1}$
Fe	$59.7^{+3.1}_{-0.9}$	$18.3^{+0.1}_{-0.1}\times10^{1}$	$7.3^{+1.8}_{-0.5}$
FeO_x	$38.6^{+1.7}_{-3.2}$	$32.4^{+1.0}_{-0.5}$	$7.9^{+0.3}_{-2.9}$
*GMO	8.9	$35.5^{+4.4}_{-34.3}$	$5.4^{+3.4}_{-2.8}$

Table S5. Initial parameter values and the bounds when fitting to the XRR data collected with the substrate in air and with a 20 mM GMO-dodecane solution entrained onto the substrate surface at 3.0×10^3 s^{-1}. * – parameters used in the GMO-dodecane contrast.

Layer	Parameter	Initial Value	Bounds	Units
Si	Thickness	∞	-	Å
	Roughness	3	-	Å
	$SLD_{X\text{-ray}}$	19.8	-	10^{-6} Å^{-2}
SiO_2	Thickness	10	(1, 30)	Å
	Roughness	5	(1, 10)	Å
	$SLD_{X\text{-ray}}$	18.6	-	10^{-6} Å^{-2}
Fe	Thickness	190	(170, 210)	Å
	Roughness	6	(1, 10)	Å
	$SLD_{X\text{-ray}}$	62.2	(55.5, 62.8)	10^{-6} Å^{-2}
FeO_x	Thickness	30	(20, 40)	Å
	Roughness	7	(1, 10)	Å
	$SLD_{X\text{-ray}}$	42.6	(31.9, 48.3)	10^{-6} Å^{-2}
GMO*	Thickness	10	(1, 40)	Å
	Roughness	7	(1, 10)	Å
	$SLD_{X\text{-ray}}$	8.9	-	10^{-6} Å^{-2}
Air	SLD_n	0.00	-	10^{-6} Å^{-2}
dodecane-h_{26}^*	SLD_n	6.42	-	10^{-6} Å^{-2}
Misc	I_0	1.013	-	-
	I_0^*	1.000	-	-
	θ-offset	0	(−0.02, 0)	degrees
